# Piezoelectric Drop-on-Demand Inkjet Printing with Ultra-High Droplet Velocity

**DOI:** 10.34133/research.0248

**Published:** 2023-10-13

**Authors:** Zhengjie Yang, Hongmiao Tian, Chunhui Wang, Xiangming Li, Xiaoliang Chen, Xiaoming Chen, Jinyou Shao

**Affiliations:** ^1^ State Key Laboratory for Manufacturing Systems Engineering, Xi’an Jiaotong University, Xi’an, Shaanxi 710049, China.; ^2^ Frontier Institute of Science and Technology (FIST), Xi’an Jiaotong University, Xi’an, Shaanxi 710049, China.

## Abstract

Improving droplet velocity as much as possible is considered as the key to improving both printing speed and printing distance of the piezoelectric drop-on-demand inkjet printing technology. There are 3 tough and contradictory issues that need to be addressed simultaneously, namely, the actuation pressure of the piezoelectric printhead, satellite droplets, and the air resistance, which seems almost impossible to achieve with classical methods. Herein, a novel solution is introduced. By modulating the positive crosstalk effect inside and outside the printhead, self-tuning can be achieved, including self-reinforcing of the actuation pressure, self-restraining of satellite droplets, and self-weakening of the air resistance, thereby greatly improving droplet velocity. Based on these mechanisms, waveform design methods for different inks and printheads are investigated. The results demonstrate that monodisperse droplet jetting with a maximum velocity of 27.53 m/s can be achieved, reaching 3 to 5 times that of the classical method (5 to 8 m/s). Correspondingly, the printing speed and distance can be simultaneously increased by almost 10 times, demonstrating an ability of direct printing on irregular surface. Meanwhile, the compatibility of ink materials is expanded, as the Ohnesorge number and the viscosity of printable inks for the printhead used are increased from 0.36–0.72 to 0.03–1.18 and from 10–12 cp to 1–40.3 cp, respectively, even breaking the traditional limitations of the piezoelectric printing technology (Ohnesorge number of 0.1 to 1; viscosity of 1 to 25 cp). All the above provide a new perspective for improving droplet velocity and may even offer a game-changing choice for expanding the boundaries of the piezoelectric drop-on-demand inkjet printing technology.

## Introduction

Piezoelectric drop-on-demand (DOD) inkjet printing, i.e., piezoelectric inkjet (PIJ) printing, is a non-contact additive manufacturing process, where droplets are ejected from a PIJ printhead and then deposited on a substrate after flying for a certain distance [1,2]. Due to the avoidance of the mechanical contact between the printhead that generates the droplets and the substrate that receives them, this brings great convenience, e.g., the material of the substrate is almost unrestricted, and the substrate can be irregular [3–6]. Thus far, the PIJ technology has been successfully applied in a wide range of fields ranging from general printing applications, e.g., graphics, textiles, packaging, ceramics, and decorations, to advanced manufacturing industries, e.g., printed electronics [7–9], biological and chemical sensors [10–12], 3-dimensional (3D) printing and additive manufacturing [13–15], and handling diverse materials including water-based inks, solvent-based inks, and polymers [16,17]. Nowadays, one of the main bottlenecks faced by the booming PIJ technology is how to improve both the printing speed (the substrate feed speed) and the printing distance.

In general, higher printing speed and longer printing distance have always been the pursuit for the researchers and practitioners of the PIJ technology [2,3,18–21]. For the printing speed, improving it involves putting up the productivity and reducing the costs, which are undoubtedly crucial for all commercial technologies, considering the benefits of improving market competitiveness. For the printing distance, improving it means stronger compatibility [22], which is one of the new engines driving the future development of the PIJ technology. Unlike traditional flat substrates, irregular substrates, e.g., rough and textured, undulating, and even freeform 3D surfaces, are becoming the major printing targets for the emerging printing needs [6,23–27]. Different from traditional 2D plane printing, where the printed flat patterns need to be accumulated in a layer-by-layer manner to form a 3D structure [28,29], printing is performed directly toward a complex target, that is, the so-called “direct to shape/object” printing [2,3,30]. This opens up opportunities for new potential applications of surface decoration/coating and surface functionalization of complex non-planar devices, e.g., “conformal electronics” [31,32], “structural electronics” [33], and “flexible hybrid electronics” [34]. Since the printing distance fundamentally limits the height fluctuation limit of the substrate, the current PIJ technology can only be used on a simple and slightly curved surface. Improving the printing distance becomes the key factor for promoting the application of “direct to shape/object” printing on irregular complex devices. Researchers have conducted detailed and systematic analyses on the factors affecting the printing speed and printing distance in PIJ, and it is believed that the limitations regarding these 2 parameters can be mainly attributed to droplet velocity [2,3].

There are 2 basic physical phenomena taking place during the droplet flying process, which are the main reasons why the printing speed and printing distance are limited by the droplet velocity. One is that the droplet velocity decelerates continuously under the effect of air resistance [22,35,36]. The position where the droplet velocity reaches a zero value is the theoretical maximum printing distance, i.e., throwing distance. Under the same conditions, the higher the droplet velocity, the longer the throwing distance. The other is that the unsteady flow near the substrate can change the droplet flying direction, even leading to imaging defects such as “wood-grain” and “fogging” [22,37–39]. This phenomenon is caused by the coupling effect between the Couette flow entrained from the substrate motion and the air flow induced from droplet drag. For a given printing speed (substrate feed speed), the higher the droplet velocity, the weaker the unstable flow caused by the coupling effect. In other words, for the same printing accuracy, the higher the droplet velocity, the higher the printing speed. Due to the limitations of the above 2 phenomena, the printing distance and printing speed of high-quality PIJ processes are now generally limited to 0.5 to 2 mm (typically 1 mm) and tens to hundreds of mm/s (typical maximum value, 500 mm/s), respectively, corresponding to the current typical droplet velocity of 5 to 8 m/s at a distance of 1 mm away from the printhead nozzle [36,40,41]. The best way to overcome the impact of the above 2 situations is to transfer sufficient energy to the jetted droplets, that is, to improve droplet velocity. The droplet velocity should be as high as possible; thus, the additional energy could be utilized to overcome any interference during the droplet flying process [2]. Improving droplet velocity would actually bring great changes. A related example is that the continuous inkjet (CIJ) technology has a droplet velocity of up to 10 to 30 m/s, and the corresponding printing distance and printing speed reach several cm and m/s [41], respectively, which are all 10 times higher than those of the current PIJ technology. Nevertheless, due to its poorer ink viscosity compatibility (generally <5 cp) and lower resolution [42], the CIJ technology is considered to be difficult to apply in the vast majority of application scenarios related to the PIJ technology. It seems to be urgent and exciting to improve droplet velocity of the PIJ technology as much as possible, even close to the level of the CIJ technology, since this may provide a game-changing choice for all PIJ-based manufacturing processes.

It is a fascinating but really challenging task. In order to greatly improve droplet velocity, 3 tough and contradictory issues need to be addressed simultaneously, namely, the actuation pressure of the PIJ printhead, satellite droplets, and the air resistance. As regards the first one, the actuation pressure of the PIJ printhead fundamentally determines the initial droplet velocity [43,44], which increases almost linearly with the increase of the actuation pressure amplitude. The ink viscosity, generally ranging within 1 to 25 cp, and the nozzle diameter of the PIJ printhead, generally ranging within 10 to 50 μm, would affect the linear proportional coefficient between actuation pressure amplitude and initial droplet velocity [4]. In general, the higher the ink viscosity and the larger the nozzle diameter, the greater the actuation pressure required to achieve the same droplet velocity. For most of the current commercial PIJ printheads, when an ink with low viscosity (<10 cp) is used, the droplet velocity could reach 10 m/s or slightly higher under the maximum actuation pressure [2,3,36]. In this case, on the one hand, such an improvement is not enough, since it would not meet the demand for high-viscosity inks (>10 cp); on the other hand, the second issue, i.e., satellite droplets, would arise. Under such a high droplet velocity, the monodisperse droplet jetting state can no longer be maintained, since the well-known but disturbing satellite droplets would occur inevitably [45], seriously reducing the printing accuracy and making it almost impossible to apply to most printing scenes. As regards the third issue, the Stokes drag in the air continuously reduces the droplet velocity throughout the entire droplet flying process [22,35,36]. The smaller the droplet volume and the farther the printing distance, the more obvious the deceleration effect, which may completely prevent droplets from reaching the substrate [13,22]. An exemplary reduction of droplet velocity in 1-mm distance for the initial droplet velocity of 10 m/s could range from 4 m/s (for a droplet diameter of 10 μm) to 0.35 m/s (for a droplet diameter of 50 μm) [2,13]. In addition, the air resistance acting on droplets is almost linearly correlated with the square of the droplet velocity [2]. In other words, the higher the droplet velocity, the higher the deceleration rate. To provide a brief overview of the above issues, the maximum droplet velocity with most of the current commercial PIJ printheads can only reach ~10 m/s even under the maximum actuation pressure. Meanwhile, along with the improvement of the droplet velocity, satellite droplets would occur inevitably and the deceleration effect induced by air resistance would become stronger, deteriorating the high droplet velocity. What a perfect paradox! Consequently, in order to achieve an ultra-high droplet velocity (UHDV) (>15 m/s, reaching the level of CIJ technology), all these 3 issues must be solved at the same time, which seems almost impossible for the classical PIJ technology.

Dramatically, in our previous research that focused on improving the frequency of the PIJ technology, experimental phenomena of monodisperse droplet jetting with velocities up to 15.5 m/s (at 117.7 kHz) and 16.7 m/s (at 133.3 kHz) were unexpectedly observed [46]. A conventional commercial piezoelectric printhead was used and the ink viscosity was 1 cp. According to the experimental results, the droplet velocity already exceeded the optimal values achieved by the available technologies. The high-frequency PIJ method seems to provide a possibility to simultaneously solve the above 3 issues of improving droplet velocity. The present article attempts to conduct systematic research on the mechanisms and methods of improving droplet velocity at high actuation frequency by combining numerical simulations and experimental methods. First, the interesting phenomenon of achieving monodisperse droplet jetting with UHDV by changing the actuation waveform is observed and discussed in detail. Second, the unrecognized self-tuning mechanisms induced by the positive crosstalk effect under high actuation frequency are revealed, providing a physical foundation for solving the above-mentioned 3 issues of improving droplet velocity. Subsequently, universal actuation waveform design methods for the UHDV-PIJ technology that could be suitable for different inks and PIJ printheads are provided. Finally, the performance of the proposed UHDV-PIJ method is assessed in terms of the compatibility of ink materials, the capabilities of high-speed and long-distance printing and the capabilities of “direct to shape/object” printing on irregular complex devices.

## Results

### The phenomenon of UHDV monodisperse droplet jetting

**Fig. 1.**
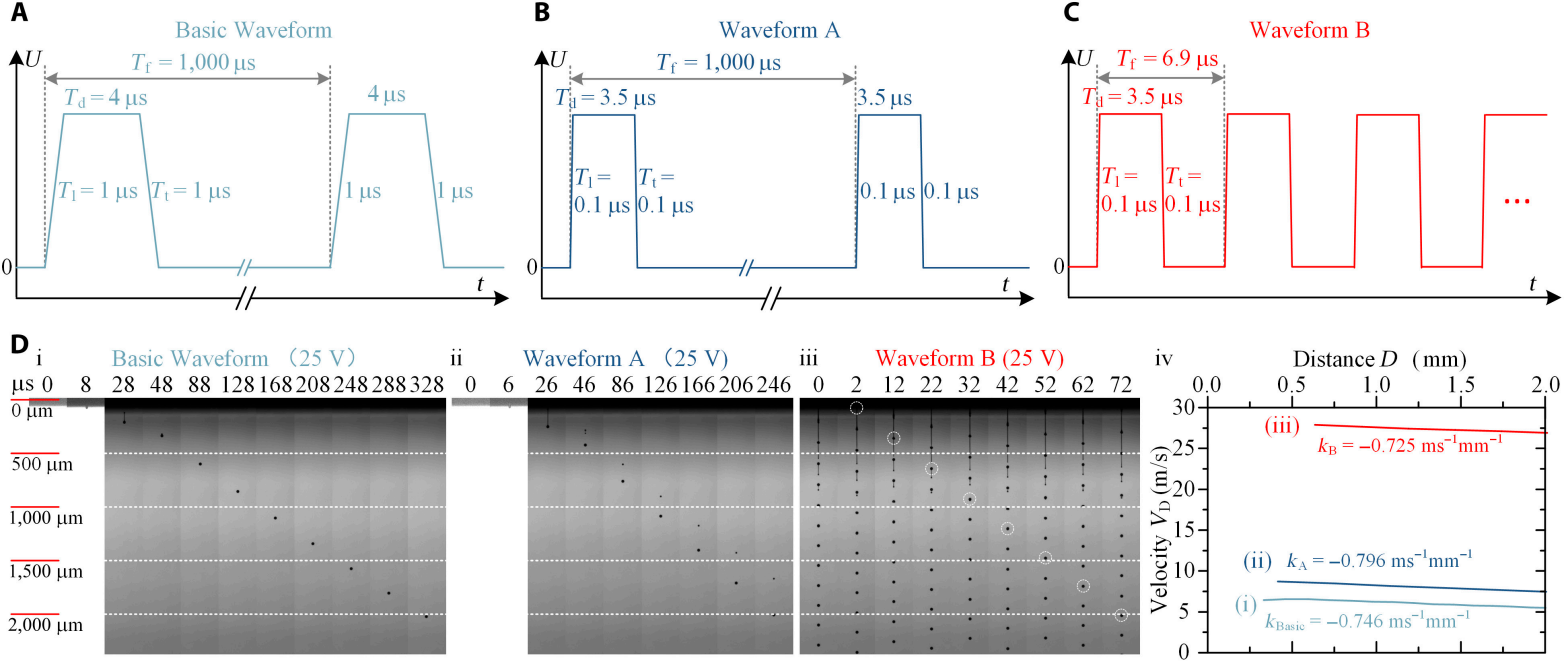
Three different actuation waveforms and corresponding experimental results. (A) Basic Waveform (1 μs–4 μs–1 μs–1 kHz). (B) Waveform A (0.1 μs–3.5 μs–0.1 μs–1 kHz). (C) Waveform B (0.1 μs–3.5 μs–0.1 μs–144.928 kHz). (D) Dynamic processes of droplet jetting under different actuation waveforms (i to iii), and corresponding droplet velocity evolution processes with increasing jetting distance (iv).

Compared to common low-frequency actuation waveforms, the phenomenon of UHDV monodisperse droplet jetting was observed in experiments under a high-frequency actuation waveform (Fig. 1).

Figure 1A to C display the 3 actuation waveforms used in the experiments, i.e., Basic Waveform (1 μs–4 μs–1 μs–1 kHz), Waveform A (0.1 μs–3.5 μs–0.1 μs–1 kHz), and Waveform B (0.1 μs–3.5 μs–0.1 μs–144.928 kHz). Basic Waveform is a commonly used low-frequency actuation waveform (recommended by the manufacturer), which served as a benchmark for comparison; Waveform A is a designed low-frequency actuation waveform; Waveform B is a designed high-frequency actuation waveform.

Figure 1D (i to iii) present experimental photographs of the droplet jetting process obtained under the 3 different waveforms, and Fig. 1D (iv) presents the evolution processes of their droplet velocity with increasing jetting distance. In all cases, the actuation voltage was 25 V and the same ink with a viscosity of 9.2 cp was used. In Fig. 1D (i to iii), there were obvious differences in the morphology and average velocity of the droplets within a distance of 2,000 μm from the nozzle. Under the Basic Waveform (Fig. 1D, i), a slender tail was generated following the main droplet in the area proximal to the nozzle, which subsequently fractured to form a satellite droplet (28 μs). The satellite droplet quickly merged with the main droplet (48 μs), and then maintained a monodisperse droplet morphology (48 to 328 μs). The average droplet velocity was equal to 6.098 m/s. Under Waveform A (Fig. 1D, ii), a slender tail with a longer length was also generated, following the main droplet in the area proximal to the nozzle, which subsequently fractured to form a satellite droplet (26 μs). However, the satellite droplet never merged with the main droplet, and the distance between the main and satellite droplets became larger and larger (26 to 246 μs). The average droplet velocity of the main droplet was equal to 8.130 m/s, i.e., 1.333 times that under the Basic Waveform. Moreover, the average droplet velocity of the satellite droplet was equal to 6.748 m/s, reaching only 83% of that of the main droplet. Under Waveform B (Fig. 1D, iii), the droplet morphology was different from that generated under the low-frequency waveform and exhibited a completely new form. It should be noted that there were many droplets within the field of view; the specific droplet that was tracked is marked with a white circle. It can be observed that jet flow with a string-of-beads style was formed near the nozzle, with the main droplets being linked together by a tail before breaking up (0 μs and 2 μs). The location where the droplet broke (12 μs) was far from the nozzle, i.e., ~500 μm way. Overall, no satellite droplet was found. The average droplet velocity of the main droplet was equal to 27.778 m/s, i.e., 3.417 times that under Waveform A.

In Fig. 1D (iv), it can be observed that there were obvious differences in the droplet velocity evolution processes with increasing jetting distance. Under the Basic Waveform (curve (i)), the velocity of the main droplet decreased gradually from 6.568 m/s to 5.503 m/s when the jetting distance was increased from 594 μm to 2,021 μm. The decay rate of the droplet velocity with jetting distance was ~0.746 ms^−1^ mm^−1^. Under Waveform A (curve (ii)), the velocity of the main droplet decreased gradually from 8.732 m/s to 7.467 m/s when the jetting distance was increased from 416 μm to 2,005 μm. The decay rate of the droplet velocity with jetting distance was ~0.796 ms^−1^ mm^−1^, i.e., 1.067 times that under the Basic Waveform. Under Waveform B (curve (iii)), the velocity of the main droplet decreased gradually from 27.833 m/s to 26.835 m/s when the jetting distance was increased from 636 μm to 2,012 μm. The decay rate of the droplet velocity with jetting distance was ~0.725 ms^−1^ mm^−1^, i.e., 0.911 times that under Waveform A. Obviously, under the high-frequency actuation waveform, the droplet velocity was greatly increased; on the contrary, the decay rate of the droplet velocity with jetting distance was suppressed.

Waveform optimization from the Basic Waveform to Waveform A resulted in a small improvement on droplet velocity (1.333 times); nevertheless, satellite droplets appeared inevitably and the air resistance increased as well (1.067 times). By increasing the actuation frequency, i.e., optimizing the waveform from Waveform A to Waveform B, the droplet velocity was greatly improved (3.417 times), while the satellite droplets were suppressed and the impact of the air resistance was weakened (0.911 times). The most noticeable change from low to high frequency is the enhancement of the crosstalk effect. Under low frequency, each jetting cycle is almost independent; however, the situation varies under high frequency, since the crosstalk effects between the pre- and post-cycles may greatly change the droplet jetting state. These crosstalk effects may mainly include the multi-pulse crosstalk effect, the interference between droplet and droplet, and the interference between droplet and air. The positive crosstalk effect may induce unrecognized self-tuning mechanisms, under which self-reinforcing of the actuation pressure of the PIJ printhead, self-restraining of satellite droplets, and self-weakening of the air resistance can be simultaneously achieved. The following task is to conduct a more detailed study on the self-tuning mechanisms induced by the positive crosstalk effect from the perspectives of the actuation pressure, satellite droplets and the air resistance.

### The self-tuning mechanisms induced by the positive crosstalk effect

#### Self-reinforcing of the actuation pressure

First, the self-reinforcing mechanism of the actuation pressure of the PIJ printhead under the influence of the positive crosstalk effect is investigated. Self-reinforcing refers to achieving reinforcement of the actuation pressure through the self-modulation of the crosstalk effect, in which the only change concerns the actuation waveform shape rather than any other external conditions. The crosstalk effect involved in this section is the multi-pulse crosstalk effect, generated by the interference between the ink inside the printhead and the solid structure of the printhead.

The actuation pressure in the pump chamber of the PIJ printhead is in the form of an oscillating pressure wave, which is generated by the deformation of the piezoelectric actuator under the excitation of an external actuation electric field. There is a special damping ratio design between the ink supply channel and the nozzle in the structural geometry of the printhead. During the positive phase of the actuation pressure, the ink in the pump chamber produces an acceleration of flowing toward the outside of the nozzle, causing the formation of a jet flow near the nozzle area. During the negative phase of the actuation pressure, the ink in the ink supply channel produces an acceleration of flowing toward the pump chamber, replenishing the ink in the pump chamber. Generally speaking, the peak of positive pressure determines the droplet velocity, while the peak of negative pressure determines whether ink can be supplied in a continuous manner, i.e., whether the droplet jetting process can be continuous. Under the same actuation waveform, the actuation pressure amplitude is almost linearly correlated with the actuation voltage. However, due to the limitations related to the characteristics of piezoelectric ceramic materials, the actuation voltage is often very limited in practical applications, resulting in a very limited actuation pressure amplitude [1,43]. Actuation waveform optimization provides an opportunity to improve the actuation pressure by modulating the multi-pulse crosstalk effect. Previous studies have reported that the multi-pulse crosstalk inside the PIJ printhead under high actuation frequency exhibits periodicity and is controlled by a constructive/destructive interference inherent mechanism [46]. In this article, the positive crosstalk effect is further expanded, i.e., the amplitude of the constructive interference is expanded, achieving self-reinforcing of the actuation pressure.

**Fig. 2.**
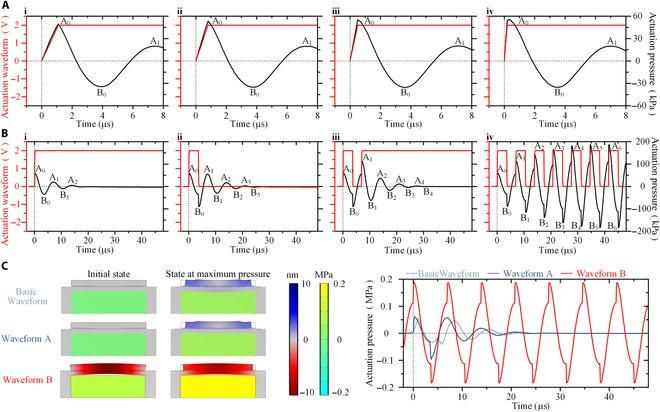
(A) Evolution process of the actuation pressure under the modulation of *T*_l_. (B) Evolution process of the actuation pressure under the modulation of *T*_d_ and *T*_f_. (C) The working pressure states and evolution curves of the pump chamber under the 3 waveforms.

As shown in Fig. 2, simulation studies have been conducted to analyze the influence of the pulse shape of the actuation waveform on the crosstalk effect and the actuation pressure. The key parameters include the leading edge time *T*_l_, pulse duration *T*_d_, and pulse period *T*_f_ (reciprocal of the pulse frequency *f*). The trailing edge time is set equal to *T*_l_.

Figure 2A shows the evolution process of the actuation pressure under the modulation of leading edge time *T*_l_. Under a waveform with a single leading edge, the actuation pressure inside the printhead reaches the first positive pressure peak quickly, and then begins to enter a residual oscillation stage, i.e., pressure crosstalk. Table S1 statistically compares the peak values and peak arrival time under different *T*_l_. When *T*_l_ is decreased from 1.1 μs to 0.2 μs, the value of the first positive pressure peak, corresponding to point A_0_ in Fig. 2A (i to iv), increases gradually from 49.54 kPa to 55.23 kPa, i.e., increased by 12%. In the subsequent pressure crosstalk stage, the values of the pressure peaks, corresponding to point B_0_ and A_1_ in Fig. 2A (i to iv), exhibit no obvious increase. Nevertheless, the peak arrival time tends to occur earlier, with *t*_B0_ advancing from 3.9 μs to 3.5 μs and *t*_A1_ advancing from 7.4 μs to 7 μs, which helps to increase the actuation frequency. Obviously, a smaller *T*_l_ is beneficial to produce a higher droplet velocity. In practice, considering the limitation of charging and discharging time of piezoelectric elements, the minimum *T*_l_ is limited to 0.1 μs.

Figure 2B presents the evolution process of the actuation pressure under the modulation of pulse duration *T*_d_ and pulse period *T*_f_. Table S2 statistically compares the peak values and peak arrival time under different waveforms. Under a waveform with a single leading edge of *T*_l_ = 0.1 μs (Fig. 2B, i), the obtained point A_0_ is (0.3 μs; 57.42 kPa), which means that the peak arrival time is 0.3 μs and the pressure peak value is 57.42 kPa. In the subsequent pressure crosstalk stage, the obtained point B_0_ is (3.6 μs; −35.31 kPa) and point A_1_ is (7 μs; 19.85 kPa). Under a waveform with a modulated pulse duration *T*_d_ (Fig. 2B, ii), where *T*_d_ is set to be equal to *t*_B0_ (obtained by Fig. 2B, i) minus *T*_l_, the actuation effect generated by the trailing edge is synchronously superimposed with the pressure crosstalk induced by the leading edge, obtaining the maximum value of the first negative pressure peak (B_0_) and pressure crosstalk (A_1_). The obtained point B_0_ is (3.9 μs; −90.25 kPa), and the value of the pressure peak is 2.56 times higher compared to that in Fig. 2B (i). In the subsequent pressure crosstalk stage, the obtained point A_1_ is (6.9 μs; 55.33 kPa), and the value of the pressure peak is 2.79 times higher compared to that in Fig. 2B (i). Under a waveform with a modulated pulse period *T*_f_ (Fig. 2B, iii), where *T*_f_ is set to be equal to *t*_A1_ (obtained by Fig. 2B, ii), the actuation effect generated by the new the leading edge of the second pulse period is synchronously superimposed with the pressure crosstalk induced by the first pulse period, obtaining the maximum value of the second positive pressure peak (A_1_) and pressure crosstalk (B_1_). The obtained point A_1_ is (7.2 μs; 112.82 kPa), and the value of the pressure peak is 2.04 times higher compared to that in Fig. 2B (ii). In the subsequent pressure crosstalk stage, the obtained point B_1_ is (10.5 μs; −62.11 kPa), and the value of the pressure peak is 2.07 times higher compared to that in Fig. 2B (ii). A comprehensive comparison of Fig. 2B (i to iii) suggests that under the modulation of pulse duration *T*_d_ and pulse period *T*_f_, the values of the positive pressure peak, negative pressure peak, and pressure crosstalk are all greatly enhanced. As presented in Fig. 2B (iv), by increasing the number of pulses, a modulation process similar to that in Fig. 2B (i to iii) will continue to take place, reaching and stabilizing at a high value state after 6 pulses (about 41.4 μs).

Figure 2C exhibits the working pressure states and evolution curves of the pump chamber under the 3 waveforms used in Fig.1. Under the Basic Waveform and Waveform A, the pressure in pump chamber and the deformation of the actuator are all zero at the initial moment before the leading edge of the waveform is applied; subsequently, the actuator begins to compress the pump chamber, generating an oscillating pressure wave. The maximum values of the positive pressure peaks are 52.74 kPa and 57.42 kPa, respectively. Under Waveform B, the pressure in the pump chamber reached 112.82 kPa at the initial moment before the leading edge of the waveform is applied, and the actuator is in an expanding state. Then, as the actuator compresses the pump chamber, the pressure in the pump chamber increases rapidly, reaching the maximum value of 186.68 kPa. The maximum value of the negative pressure peaks is −183.89 kPa.

By comparing the positive pressure peak values under the 3 different waveforms, it can be seen that the maximum value increased by 1.09 times from Basic Waveform to Waveform A, and by 3.25 times from Waveform A to Waveform B. These basically match the experimental results in Fig.1, and the self-reinforcing mechanism of the actuation pressure under the influence of the positive crosstalk effect has been well explained and verified. In addition, as regards PIJ printheads with other structural forms, e.g., shear mode, push mode, and squeeze mode, simulation results indicate that the actuation pressures are usually increased by 2 to 4 times (Fig. S1), suggesting that the self-reinforcing mechanism of the actuation pressure proposed in this article is universal for different PIJ printheads.

In addition, the self-reinforcing phenomenon similar to Fig. 2 may also be obtained at different frequencies, as shown in Fig. S2. When only considering the frequency, as the jetting frequency gradually increases from 1 kHz to 144.928 kHz, the actuation effect generated by the next leading edge will be superimposed with the previous pressure crosstalk at a different position (Fig. S2A), theoretically resulting in the phenomenon of the droplet jetting state oscillating regularly with the increase of the pulse frequency [46]. The supplementary experimental results (Fig. S2B) at different frequencies match the theory well. The droplet jetting state is stable at a jetting frequency of superimposing near the positive peak point of pressure crosstalk, such as 144.928 kHz (6.9 μs), 166.667 kHz (6 μs), 133.333 kHz (7.5 μs), 72.464 kHz (13.8 μs), and 48.309 kHz (20.7 μs). By contrast, nothing could be observed at a jetting frequency of 100 kHz (10 μs), where the actuation effect is superimposed near the negative peak point of pressure crosstalk. As shown in Fig. S2C and D, the modulation of the pressure crosstalk inside the printhead can be adjusted by adjusting the actuation pulse waveform parameters, such as leading edge time *T*_l_, pulse duration *T*_d_, and trailing edge time *T*_t_, to achieve stable droplet jetting at any desired frequency [46]. One case is that droplet jetting cannot be performed at 100 kHz under the waveform of 0.1 μs–3.5 μs–0.1 μs, while a stable droplet jetting state is achieved at 100 kHz under the waveform of 1 μs–5 μs–1 μs (Fig. S2C). Another case is that the limit jetting frequency for stable jetting is 166.667 kHz (6 μ s) under the waveform of 0.1 μs–3.5 μs–0.1 μs, while the limit jetting frequency for stable jetting is 181.818 kHz (5.5 μs) under the waveform of 0.3 μs–1.3 μs–0.7 μs (Fig. S2D). The Helmholtz oscillation period *T*_o_ of the printhead system itself (equals to *t*_A2_ − *t*_A1_ in Fig. 2B, ii), which is mainly influenced by the printhead design and the viscosity of the ink, ultimately determines the limit jetting frequency. In general, for a waveform with a given leading edge time *T*_l_, pulse duration *T*_d_, and trailing edge time *T*_t_, at its limit jetting frequency, the period of the waveform *T*_f_ (i.e., the reciprocal of the frequency) is approximately equal to the sum of the pulse length (*T*_l_ + *T*_d_ + *T*_t_) and 1/2 *T*_o_ [46]. Based on experimental results and theoretical estimation, the stable droplet jetting at any frequency within 181.818 kHz (5.5 μs) could be achieved for the used ink with a viscosity of 9.2 cp. As shown in Fig. S2E, the variation of droplet velocity with increasing actuation voltage at different frequencies are analyzed, considering that the core of this work is to achieve stable droplet jetting with UHDV. Obviously, only when the actuation effect is synchronously superimposed with the pressure crosstalk (Fig. 2B), i.e., 144.928 kHz (6.9 μs), is the self-reinforcing effect of the actuation pressure most strong and has the droplet velocity the most sensitive response to the actuation voltage, making it easiest to achieve droplet jetting with UHDV. At other frequencies, the self-reinforcing effect of the actuation pressure will be weakened, and a larger actuation voltage is required to achieve the same droplet velocity.

#### Self-restraining of satellite droplets

Second, the self-restraining mechanism of satellite droplets under the influence of the positive crosstalk effect is investigated. Self-restraining refers to eliminating the impact of satellite droplets on printing quality through the self-modulation of the crosstalk effect. In this case, satellite droplets actively merge with subsequent main droplets during the droplet flying process, without any other external conditions. The crosstalk effect involved in this part is the interference between droplet and droplet.

**Fig. 3.**
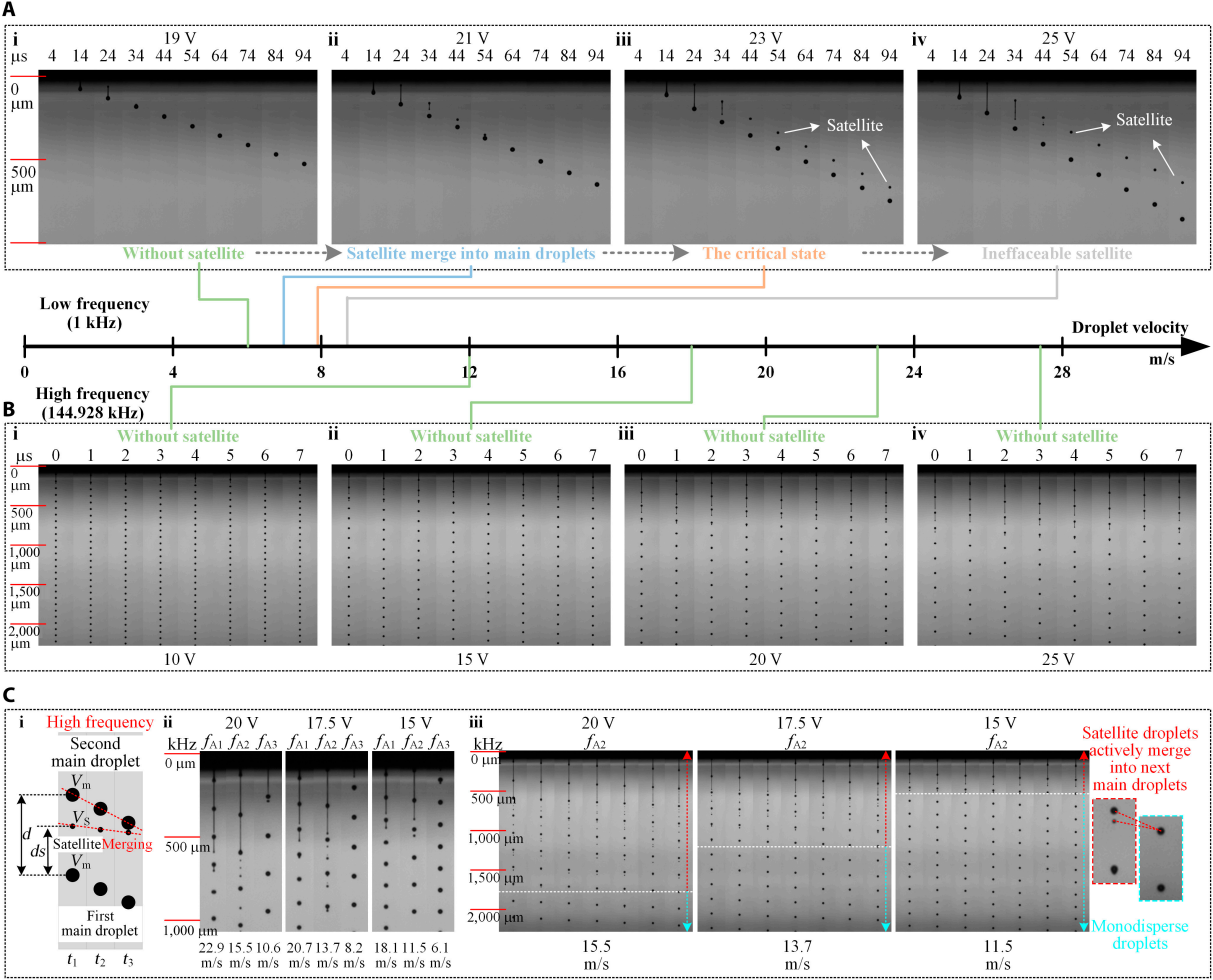
The generation of satellite droplets and the self-restraining phenomena. Change of droplet morphology with increasing droplet velocity at (A) low frequency and (B) high frequency. (C) Mechanism of eliminating satellite droplets under high frequency.

As depicted in Fig. 3, the generation law of satellite droplets and the self-restraining phenomena are discussed. Figure 3A compares the change of droplet morphology with increasing droplet velocity under low frequency. In Fig. 3A (i to iv), under low frequency (Waveform A; 1 kHz), 4 different droplet morphology types appear with increasing droplet velocity, including without satellite droplets (Fig. 3A, i), satellite droplets merge with the main droplets (Fig. 3A, ii), the critical state (Fig. 3A, iii), and ineffaceable satellite droplets (Fig. 3A, iv). Under the type in Fig. 3A (i), the droplet velocity is about 5.83 m/s at 0.5 mm at an actuation voltage of 19 V and droplet jetting maintains a monodisperse state (34 to 94 μs) without the appearance of satellite droplets. Under the type in Fig. 3A (ii), the droplet velocity is about 7.08 m/s at 0.5 mm at an actuation voltage of 21 V and 2 satellite droplets are formed (34 μs); however, they quickly merge into the main droplet (34 to 54 μs) and then maintain the monodisperse droplet morphology (64 to 94 μs). Under the type in Fig. 3A (iii), the droplet velocity is about 7.92 m/s at 0.5 mm at an actuation voltage of 23 V and many satellite droplets are formed (34 μs), which merge into a larger satellite droplet (44 μs) that has a velocity close to that of the main droplet. For a long time to come (54 to 94 μs), this satellite droplet maintains the same distance from the main droplet and does not fade way. Under the type in Fig. 3A (iv), the droplet velocity is about 8.67 m/s at 0.5 mm at an actuation voltage of 25 V and many satellite droplets are also formed (34 μs), which also merge into a larger satellite droplet (54 μs); nevertheless, the velocity of the satellite droplet is lower than that of the main droplet, resulting in an increasing distance between the main droplet and the satellite droplet (54 to 94 μs).

In Fig. 3B, under high frequency (Waveform B; 144.928 kHz), droplet jetting maintains monodisperse states with increasing droplet velocity, without the appearance of satellite droplets. The droplet velocity is 11.91 m/s at 1 mm (Fig. 3B, i), 18.14 m/s at 1 mm (Fig. 3B, ii), 22.94 m/s at 1 mm (Fig. 3B, iii), and 27.53 m/s at 1 mm (Fig. 3B, iv), corresponding to actuation voltages of 10 V, 15 V, 20 V, and 25 V. In all cases, there are tails in front of and behind the main droplet, with the length of the tail increasing with increasing actuation voltage. However, under high frequency, the morphology of the formed droplets is almost opposite to that of droplets formed at a low frequency; that is, there exists a small droplet at the head and a large droplet at the end, connected with a tail in the middle (Fig. S3). The reason for this phenomenon is that the tail and satellite droplets generated by the fracture of the first main droplet are quickly merged with the subsequent main droplets. This process is so rapid that there is no chance to see the formation of any individual satellite droplets. The high frequency of droplet jetting is the reason behind the occurrence of this phenomenon.

In Fig. 3C, a more in-depth discussion was conducted on the mechanism of eliminating satellite droplets under high frequency. Figure 3C (i) illustrates a schematic diagram of the process of satellite droplets being actively merged with subsequent main droplets during the droplet flying process, where *f* is the droplet jetting frequency, *d* is the distance between the first and second main droplets, *V_S_* is the velocity of the satellite droplet, *V_m_* is the velocity of the main droplet, and *d_S_* is the distance between a satellite droplet and the first main droplet. Assuming that *d* is relatively small, the difference of the velocity between the first and second main droplets can be ignored. If the elapsed time after a satellite droplet separates from the first main droplet is *t*, the relationship between the above parameters can be expressed as:

$$d=V_{m}\times \frac{1}{f}$$ (1)

$$d_{S}=\int_{0}^{t}\left(V_{m}-V_{S}\right)\mathrm{dt}$$ (2)

When *d_S_* is equal to *d*, the center of the satellite droplet coincides with that of the subsequent main droplet, thus eliminating the impact of satellite droplets on printing quality. Assuming that the corresponding time is *T_ms_*, an approximate equivalence relationship can be obtained:

$$V_{m}\times \frac{1}{f}=\int_{0}^{T_{\mathrm{ms}}}\left(V_{m}-V_{S}\right)\mathrm{dt}$$ (3)

The key parameter is *T_ms_*, which determines the merging state. In general, the larger the *T_ms_*, the longer the satellite droplet will exist, and vice versa. When *T_ms_* is large enough, the satellite droplet could not merge with the subsequent main droplet within the printing distance, which would affect the printing quality; when *T_ms_* is small enough, there is not enough time to see the formation of satellite droplet, just as seen in Fig. 3B (i to iv).

Considering that the volume of the main droplet is larger than that of the satellite droplet, it can be approximately assumed that, for the same droplet flying duration, the decrease in velocity of the main droplet will be smaller than that of the satellite droplet [22]. Therefore, assuming that *V_m_* and *V_s_* remain constant during the droplet flying process, an approximate value of *T_ms_* slightly larger than the actual value will be obtained:

$$T_{\mathrm{ms}}=\frac{V_{m}}{V_{m}-V_{S}}\times \frac{1}{f}=\frac{1}{1-\frac{V_{S}}{V_{m}}}\times \frac{1}{f}$$ (4)

*T_ms_* mainly depends on the ratio of the satellite droplet velocity to the main droplet velocity *V_S_/V_m_* and on the droplet jetting frequency *f*. When *V_S_/V_m_* is close to 1, *T_ms_* is close to infinity, which means that the satellite droplet will never merge with the subsequent main droplet. This situation usually occurs in the CIJ technology, where a constant-pressure source is used to accelerate the ink [42]. Thus, the velocity of the satellite droplet is naturally close to that of the main droplet, and external conditions are needed to assist in changing the direction of the satellite droplet, thereby eliminating its impact on printing quality. In the PIJ technology, *V_s_* is always not equal to *V_m_*, due to the use of a pulse pressure source. The value of *V_S_/V_m_* changes with the actuation voltage. The smaller the *V_S_/V_m_* and the higher the jetting frequency, the shorter the time needed for the satellite droplet to be actively merged with the subsequent main droplet. In Fig. 3C (ii and iii), experiments were conducted under different frequencies (*f*_A1_, *f*_A2_, and *f*_A3_ correspond to the actuation frequencies obtained at points A1, A2, and A3 in Fig. 2B, where approximately *f*_A1_ = 2*f*_A2_ = 3*f*_A3_) and different droplet velocities (obtained at 20 V, 17.5 V, and 15 V) to verify the effectiveness of the theory described as Eq. 4. Under *f*_A1_ and *f*_A3_, the value of *V_S_/V_m_* is small, and the satellite droplets are quickly eliminated. Under *f*_A2_, the value of *V_S_/V_m_* is larger, and longer times are needed. Under the actuation voltage of 15 V, *V_S_/V_m_* is close to 8/15, and the value of *T_ms_* calculated according to Eq. 4 is 2.1/*f*_A2_, which matches the *T_ms_* of 1/*f*_A2_ − 2/*f*_A2_ obtained experimentally. Under the actuation voltage of 17.5 V, *V_S_/V_m_* is close to 7/9, and the value of *T_ms_* calculated according to Eq. 4 is 4.5/*f*_A2_, which matches the *T_ms_* of 4/*f*_A2_ − 5/*f*_A2_ obtained experimentally. Under the actuation voltage of 20 V, *V_S_/V_m_* is close to 4/5, and the value of *T_ms_* calculated according to Eq. 4 is 5/*f*_A2_, which matches the *T_ms_* of 5/*f*_A2_ − 6/*f*_A2_ obtained experimentally.

In theory, as long as the flying distance is long enough, satellite droplets will eventually be eliminated. The value of *V_S_/V_m_* is affected by many factors; therefore, in practical applications, increasing the jetting frequency *f* is the most practical and convenient method to realize self-restraining of satellite droplets.

#### Self-weakening of the air resistance

Third, the self-weakening mechanism of the air resistance under the influence of the positive crosstalk effect is investigated. Air resistance refers to the frictional force that droplets experience during the flying process in the air, which will reduce droplet velocity. Self-weakening refers to weakening the deceleration effect on droplet velocity through the self-modulation of the crosstalk effect. In this case, the air resistance of the droplet can be weakened by the shielding effect caused by the accelerated air flow induced by the moving droplet in front, without any other external conditions. The crosstalk effect involved in this part concerns the interference between droplet and air.

**Fig. 4.**
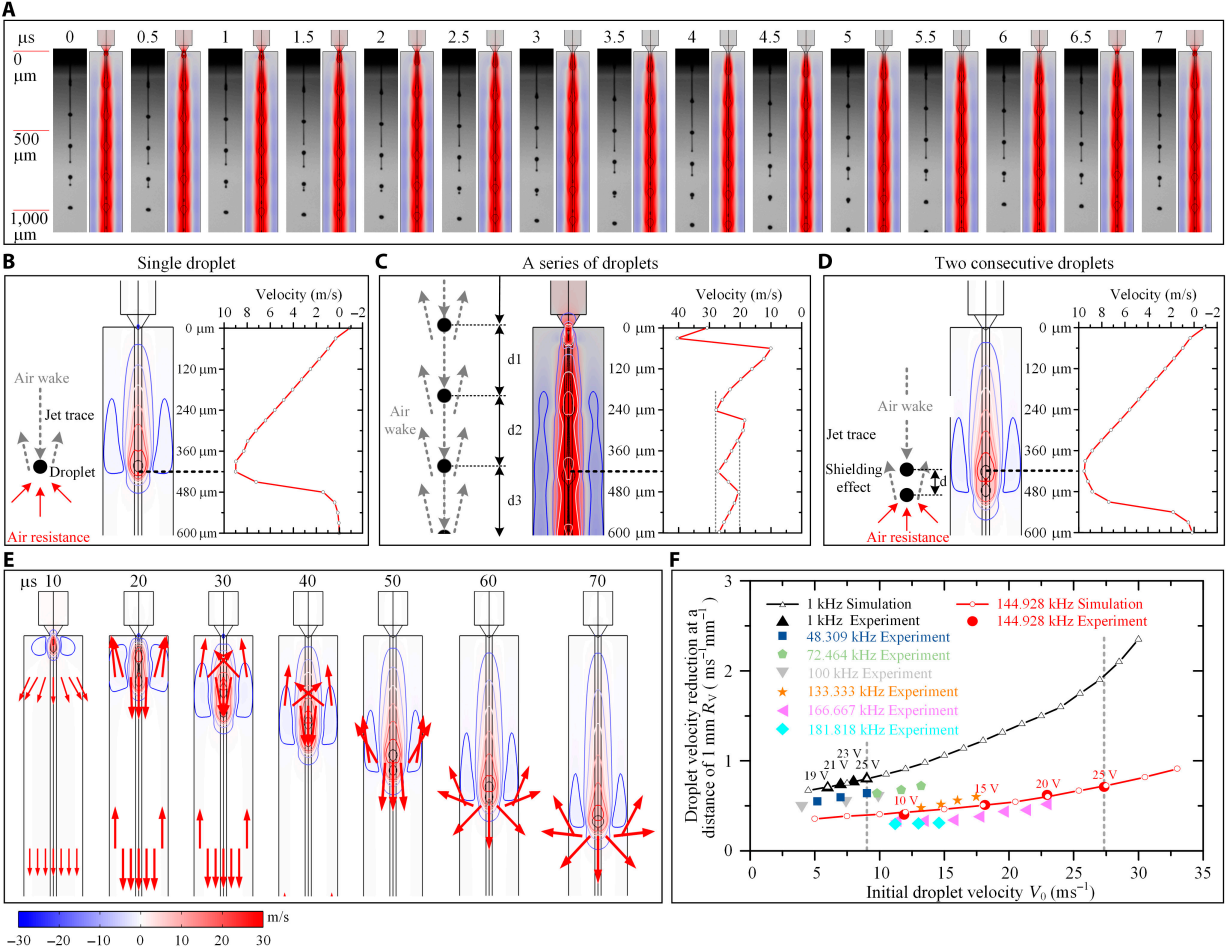
Deceleration effect of droplet velocity caused by air resistance under different jetting conditions: (A) Comparison between simulation and experimental results of the droplet jetting process under a high-frequency waveform. Distribution and evolution process of the droplet velocity at (B) low frequency and (C) high frequency under 2 consecutive droplets (D). (E) Droplet flying process of the 2 consecutive droplets. (F) Air resistance under different initial droplet velocities at low and high frequencies.

As shown in Fig. 4, the deceleration effect of the droplet velocity caused by air resistance under different jetting conditions is discussed. Figure 4A compares the simulation and experimental results of the droplet jetting process under a high-frequency waveform (Waveform B; 144.928 kHz) and an actuation voltage of 25 V. The position and velocity distribution of droplets obtained by simulation and experiments matched very well, proving the effectiveness of the simulation. The only obvious difference lies in the morphology of the satellite droplets, which is attributed to the insufficient refinement of the mesh. The size of the satellite droplet is only about 1/3 to 1/10 times that of the main droplet; thus, more refined meshes are needed, i.e., an increase of 3 to 10 times to the number of meshes, which would result in excessive computational complexity. To this end, the satellite droplets were ignored in the simulation. Figure 4B and C compare the distribution and evolution process of droplet velocity at low and high frequencies. In Fig. 4B, under low frequency (Waveform A; 1 kHz; 25 V), an approximately single-droplet jetting state is obtained, with a droplet velocity of 9 m/s at 420 μm. As it can be seen within 420 to 480 μm, the flying droplet experiences air resistance in the head area, the air forces the droplet to decelerate, and the droplet pushes the air in front of it to generate forward flow; the velocity of the air is higher than 0. On the jet trajectory, the droplet drags the air behind it, generating an airflow in the same direction. As it can be observed in the area of 0 to 380 μm, the closer the position to the droplet, the higher the velocity of the induced airflow. In Fig. 4C, under high frequency (Waveform B; 144.928 kHz; 25 V), a series of droplets are observed simultaneously, with a droplet velocity of 27.5 m/s at 420 μm. In the stable droplets train, the internal airflow velocity is also accelerated to 20 m/s, as seen in the area of 240 to 600 μm, which is close to 73% of the droplet velocity. The coupling between fast airflow and fast droplets greatly reduces the air resistance experienced by the droplets.

Figure 4D and E provide a more intuitive interpretation of the coupling between airflow and droplets by showcasing the distribution and evolution process of the droplet velocity of 2 consecutive droplets. The 2 consecutive droplets are jetted by 2 identical actuation pulses with a pulse period of 6.9 μs, which have the same shape with the actuation pulse in Waveform A. In Fig. 4D, the droplet velocity of the second droplet is 9.5 m/s at 420 μm. However, when reaching the same position, the droplet velocity of a single droplet is only 9.0 m/s at 420 μm (Fig. 4B); this indicates that the attenuation of the droplet velocity of the second droplet is weakened. More specifically, the air between the 2 consecutive droplets is simultaneously dragged by the first droplet and pushed by the second one, generating an airflow with a velocity close to the droplet velocity. This indicates that a shielding effect of air resistance is applied on the second droplet. In Fig. 4E, the droplet flying process of the 2 consecutive droplets is displayed. The attenuation of the droplet velocity of the first droplet is larger than that of the second droplet, and the distance between them decreases gradually until they eventually merge (30 to 70 μs), which provides a more intuitive demonstration of the shielding effect.

To further evaluate the shielding effect, Fig. 4F compares the air resistance under different initial droplet velocities *V*_0_ for the frequency of 1 kHz (Fig. 3A), 48.309 kHz (Fig. S2B), 72.464 kHz (Fig. S2B), 100 kHz (Fig. S2C), 133.333 kHz (Fig. S2B), 144.928 kHz (Fig. 3B and Fig. S2B), 166.667 kHz (Fig. S2B), and 181.818 kHz (Fig. S2D). The droplet velocity reduction at a distance of 1 mm, denoted as *R_V_*, is proportional to the amount of air resistance experienced by the droplet; therefore, it is used to characterize the air resistance. Theoretically, the air resistance on a droplet is determined by both droplet velocity and droplet jetting frequency. At the same droplet velocity, if the number of the flying droplets is the same, the air resistance is smaller for the droplet with larger frequency. The reason is that at the same droplet velocity, the larger the frequency, the smaller the distance between adjacent droplets and the relative velocity between the droplet and its front air, resulting in a smaller air resistance. The simulation and experimental results showed that under the same droplet velocity, the air resistance will decrease with the increase of the drop jet frequency, further proving that the air resistance with larger frequency is smaller.

Under low frequency (Waveform A; 1 kHz), i.e., the black line in Fig. 4F, the relationship between initial droplet velocity *V*_0-low_ and droplet velocity reduction at a distance of 1 mm, i.e., *R_V-low_*, can be approximated as:

$$R_{V-\mathrm{low}}=a_{1}+b_{1}\times V_{0-\mathrm{low}}^{x1}$$ (5)

Under high frequency (Waveform B; 144.928 kHz) i.e., the red line in Fig. 4F, the relationship between initial droplet velocity *V*_0-high_ and droplet velocity reduction at a distance of 1 mm, i.e., *R_V-high_*, can be approximated as:

$$R_{V-\text{high}}=a_{2}+b_{2}\times V_{0-\text{high}}^{x2}$$ (6)

where *a*_1_, *b*_1_, *x*1, *a*_2_, *b*_2_, and *x*2 are constants. According to the change trend of the curves in Fig. 4F, *x*1 is larger than *x*2, which suggests that *R_V-low_* increases faster than *R_V-high_*. In other words, the air resistance under high frequency increases slower than that under low frequency. For instance, at an initial droplet velocity of 27.7 m/s, *R_V-low_* is 1.955 ms^−1^ mm^−1^, while *R_V-high_* is 0.725 ms^−1^ mm^−1^ (only 37% of *R_V-low_*). This indicates that, for the same initial droplet velocity, the theoretical droplet flying distance at high frequency (Waveform B; 144.928 kHz) will be ~2.7 times higher than that at low frequency (Waveform A; 1 kHz).

Obviously, increasing the jetting frequency *f* helps to form an obvious shielding effect and achieve self-weakening of the air resistance during the droplet flying process.

#### Limitations of the start and stop processes

Finally, considering that the mechanisms of self-reinforcing of the actuation pressure, self-restraining of satellite droplets, and self-weakening of air resistance at the start and stop processes may differ from the stable droplet jetting process in the actual pattern printing process, experimental evaluations (Fig. S4) were conducted on the droplet jetting states of the start and stop processes.

As shown in Fig. S4A, a special waveform was designed to observe the droplet jetting state at the start and stop processes. At the front of the waveform (0 to 0.69 ms), the setting is exactly the same as Waveform B, ensuring that 1,000 droplets are jetted exactly the same as Waveform B in each waveform period. The back of the waveform (0.69 ms to 10 ms) is the idle time, and the waveform period is 10 ms (frequency of 0.1 kHz). The droplet jetting processes in each waveform period are repeated, making the start and stop processes to be cleverly captured by the droplet observation module. The actuation voltage is 22 V.

During the start process, the velocities of the first 5 droplets were lower than that of the subsequent droplets, as the actuation pressure in the first 5 actuation periods were lower than that of the subsequent actuation periods (Fig. 2B, iv). As shown in Fig. S4B and C, the front droplet is constantly overtaken by the subsequent droplets during the flying process at the start stage, merging into a larger “First Main Droplet”. The droplets formed during the start stage, i.e., the stage before the “First Main Droplet” reaches the substrate, cannot guarantee the accuracy of pattern printing. The time of the start stage is usually related to the printing distance, e.g., the time is 85 μs at a printing distance of *D* = 1 mm (Fig. S4B) and the time is 145 μs at a printing distance of *D* = 2 mm (Fig. S4C). Within a typical printing distance of several millimeters or even tens of millimeters, the start stage takes several tens of microseconds to a few milliseconds, which is quite brief and has a negligible impact on printing efficiency.

During the stop process, the residual pressure crosstalk and satellite droplet may have an influence on the printing process. After the droplets are ejected from the printhead, there will still be residual pressure crosstalk inside the printhead, such as point A_1_ in Fig. 2A and B (i and ii). Under the proposed UHDV-PIJ method, the pressure inside the printhead is very high, resulting in obvious residual pressure crosstalk, even to the level of producing “Residual Droplet” (Fig. S4B and C). In addition, after the “Last Main Droplet” (Fig. S4B and C) is ejected, there are no subsequent droplets to achieve the self-restraining of satellite droplets, thus generating the “Last Satellite Droplets” (Fig. S4B and C). The time of the stop stage also equals to several tens of microseconds to a few milliseconds, and the impact on printing efficiency is also minimal.

As shown in Fig. S4D, in order to eliminate the impact of “First Main Droplet”, “Residual Droplet”, and “Last Satellite Droplets” on printing quality, simple shielding masks should be added outside the substrate to filter out the droplets generated during the start and stop stages.

### Actuation waveform design of UHDV-PIJ for different inks and printheads

**Fig. 5.**
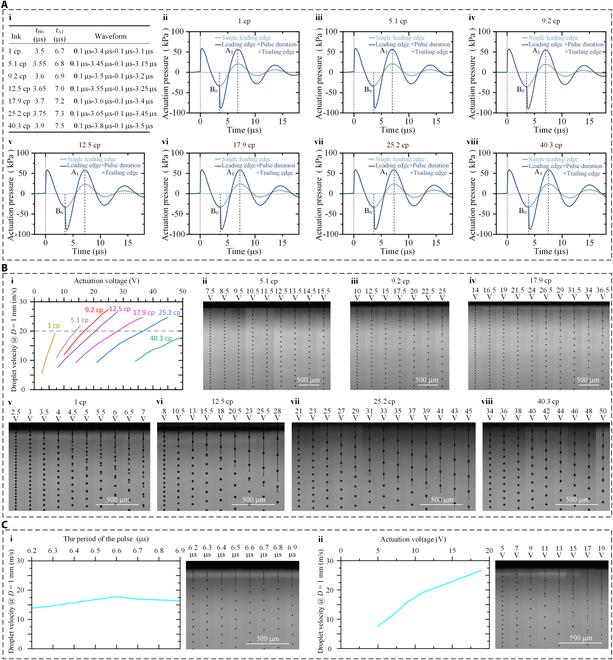
(A) (i) Actuation waveforms for 7 different inks; (ii to viii) pressure oscillation curves inside the printhead obtained by simulation under the 7 different waveforms. (B) (i) Droplet velocity variation with increasing voltage under different viscosities; (ii to viii) experimental photographs of the droplet jetting states obtained under different actuation voltages and 7 different ink viscosities. (C) Experimental results of droplet velocity and droplet jetting state obtained (i) under waveforms with different *T*_f_ and (ii) under the waveform of 0.1 μs–3.3 μs–0.1 μs–3.1 μs with increasing actuation voltage.

Considering that the self-reinforcing of the actuation pressure, self-restraining of satellite droplets, and self-weakening of the air resistance can all be achieved under high-frequency actuation waveforms through the modulation of the positive crosstalk effect, a direction for achieving UHDV-PIJ with different materials and different printheads is provided. As shown in Fig. 5, actuation waveform design methods for different inks and different printheads are discussed.

In Fig. 5A and B, a simulation-based UHDV-PIJ actuation waveform design method is demonstrated. As depicted in Fig. 5A, numerical simulations provide a low cost and effective method for developing and predicting new UHDV-PIJ actuation waveforms, guiding by the following design guidelines: minimization of the leading edge time *T*_l_, *T*_d_ is set to be equal to *t*_B0_ − *T*_l_, and *T*_f_ is set to be equal to *t*_A1_. Based on the above guidelines, *T*_l_ is set to be equal to 0.1 μs, and the actuation waveforms obtained for different inks with viscosities of 1.0 cp, 5.1 cp, 9.2 cp, 12.5 cp, 17.9 cp, 25.2 cp, and 40.3 cp are displayed in Fig. 5A (i). The corresponding acquisition processes of *T*_d_ and *T*_f_ are depicted in Fig. 5A (ii to viii). Moreover, *t*_B0_ is determined by the pressure oscillation curve under a waveform with a single leading edge, and *t*_A1_ is determined by the pressure oscillation curve under a waveform with a modulated pulse period, including the leading edge, pulse duration, and the trailing edge. It can be observed that, when the ink viscosity increases from 1 cp to 40.3 cp, *t*_B0_ and *t*_A1_ increase gradually as well. In particular, *t*_B0_ increases from 3.5 μs to 3.9 μs, *t*_A1_ increases from 6.7 μs to 7.5 μs, and the corresponding actuation frequency decreases from 149.254 kHz to 133.333 kHz. This is mainly caused by the fluid–structure coupling mechanisms between the solid structure and the ink inside the printhead. On the one hand, the actuation pressure provided by the piezoelectric actuator needs to squeeze the ink in the nozzle to form droplets, while the viscous load generated at the nozzle is linearly related to the ink viscosity. On the other hand, the viscous load generated at the fluid–structure interface also changes with the ink viscosity. The change of the ink viscosity changed the oscillation period of the piezoelectric actuator and the pressure inside the printhead, thus shifting the positions of points B_1_ and A_1_, resulting in the modification of the frequency required for realizing the self-reinforcing of the actuation pressure. Obviously, the pressure waves inside the printhead are delayed with increasing ink viscosity, which results in a decrease in the frequency of the corresponding actuation waveforms.

As depicted in Fig. 5B, the experimental results of the droplet velocity and droplet jetting state obtained with increasing actuation voltage under the designed waveforms for different ink viscosities are presented. In the experiments, each type of ink was tested using a different brand-new printhead to eliminate the presence of residual ink that may have an impact on ink performance. Figure 5B (i) compares the variation of droplet velocity with increasing voltage under different viscosities. The obtained maximum droplet velocity reached 19.35 m/s under 1 cp (7 V), 21.92 m/s under 5.1 cp (15.5 V), 27.53 m/s under 9.2 cp (25 V), 26.57 m/s under 12.5 cp (28 V), 24.67 m/s under 17.9 cp (36.5 V), 24.66 m/s under 25.2 cp (45 V), and 17.80 m/s under 40.3 cp (50 V). When the ink viscosity is increased, the required actuation voltage increases as well, and the rate of the change of droplet velocity with respect to the actuation voltage decreases (e.g., from 3.08 ms^−1^ V^−1^ under 1 cp to 0.51 ms^−1^ V^−1^ under 40.3 cp), which is consistent with the classical PIJ methods [1]. Meanwhile, under the same viscosity, the rate of the change of the droplet velocity with respect to the actuation voltage also decreases, especially when the droplet velocity is high. This phenomenon occurs due to the nonlinear increase of the jetting resistance with increasing droplet velocity, especially the inertial and viscous resistance [1]. In Fig. 5B (ii to viii), experimental photographs of the droplet jetting states obtained under different actuation voltages and ink viscosities are displayed. It can be observed that, when the actuation voltage is increased, the droplet velocity increases while maintaining the monodisperse droplet jetting state, proving the effectiveness of the designed actuation waveforms. Meanwhile, at the maximum actuation voltage for different inks, i.e., 1 cp (7 V), 5.1 cp (15.5 V), 9.2 cp (25 V), 12.5 cp (28 V), 17.9 cp (36.5 V), 25.2 cp (45 V), and 40.3 cp (50 V), the experimental results of droplet jetting processes are very stable, as shown in Fig. S5. This indicates that the value of the negative pressure peak inside the printhead still does not exceed the ultrasonic cavitation threshold [47–49] of the ink even if the jetting frequency is as high as 149.254 kHz to 133.333 kHz; thus, no ultrasonic cavitation occurs.

In Fig. 5C, an experimental-based UHDV-PIJ actuation waveform inverse design method is presented, which can be mainly used for the waveform design of printheads with an unknown structure. The DMC-11601 printhead with a nozzle diameter of 9 μm was selected for the validation experiments. The selected printhead basically has the minimum nozzle diameter of the current commercial PIJ printheads. An ink with a viscosity of 12.5 cp is used. According to the characteristics of the waveforms in Fig. 5A, the pulse duration *T*_d_ in the waveform design process is analogously set to half of the pulse period *T*_f_; i.e., when the pulse period is *T*_f_, the actuation waveform is 0.1 μs–(0.5*T*_f_) μs–0.1 μs–(0.5*T*_f_ -0.2) μs. In the classical PIJ method (waveform of 1 μs–4 μs–1 μs–1 kHz), the monodisperse droplet jetting state could be obtained under an actuation voltage of 20 to 30 V. An actuation voltage of 10 V was selected as reference for the experimental exploration, considering that the actuation pressure for printheads with different structures could generally be increased by 2 to 4 times under the UHDV-PIJ method (Fig. 2 and Fig. S1). Figure 5C (i) demonstrates the experimental results regarding the droplet velocity and droplet jetting state obtained under different waveforms with a *T*_f_ of 6.2 μs, 6.3 μs, 6.4 μs, 6.5 μs, 6.6 μs, 6.7 μs, 6.8 μs, and 6.9 μs. In all cases, the actuation voltage was all set to 10 V. The maximum droplet velocity of 17.83 m/s was obtained when *T*_f_ is equal to 6.6 μs. Thus, the optimal waveform for the DMC-11601 printhead is set to that obtained when *T*_f_ is equal to 6.6 μs, i.e., 0.1 μs–3.3 μs–0.1 μs–3.1 μs. In Fig. 5C (ii), the experimental results regarding the droplet velocity and droplet jetting state obtained under the waveform of 0.1 μs–3.3 μs–0.1 μs–3.1 μs with increasing actuation voltage are displayed. The obtained droplet velocity reached 7.64 m/s under 5 V, 11.46 m/s under 7 V, 15.92 m/s under 9 V, 19.10 m/s under 11 V, 21.01 m/s under 13 V, 22.92 m/s under 15 V, 24.83 m/s under 17 V, and 26.74 m/s under 19 V. The growth trend of droplet velocity exhibited a relatively apparent change at 11 V with increasing voltage, which can be mainly attributed to the large change of temperature in the laboratory (25 °C [± 2 °C]). Nonetheless, this problem does not occur in industrial production, where the temperature of the printhead is generally strictly controlled by an expensive and dedicated temperature control system. Based on the trend of satellite droplets being merged with the main droplet under the voltage of 11 V, 13 V, 15 V, and 17 V, it can be inferred that satellite droplets would eventually merge with the main droplet at a location outside the field of view of the photograph under the voltage of 19 V.

All the above results prove that the proposed simulation-based waveform design method and experimental-based waveform reverse design method are all feasible and effective, which can greatly reduce the process difficulty and cost, enabling the rapid waveform design for different inks and printheads. Maximum droplet velocity for inks with a viscosity ranging from 5.1 to 25.2 cp reaches 27.53 m/s. Even for inks with a viscosity of 40.3 cp and 1 cp, which are difficult to jet or to control the generation of satellite droplets using the classical inkjet printing methods, the maximum droplet velocity still reaches 17.80 m/s and 19.35 m/s, respectively. Correspondingly, in the current PIJ method, the monodisperse droplet jetting state could only be achieved under a droplet velocity of 5 to 8 m/s, for the inks with a viscosity range of 1 to 25 cp [2,3]. It should be highlighted that the optimal viscosity recommended by the manufacturer for the printhead used in this article is about 10 to 12 cp. By comparison, it would be no exaggeration to say that this is a transformative improvement.

### Performance comparison with existing technology

**Fig. 6.**
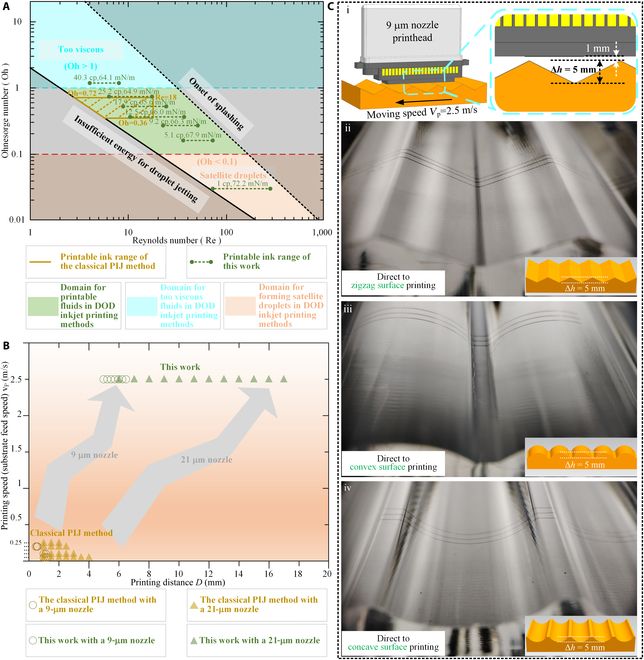
(A) Comparison of the printable ink range in the parameter space of *Re* and *Oh*. The green domain represents the domain for printable inks, the blue domain represents the domain for too viscous inks, the orange domain represents the domain for forming satellite droplets, the black solid line represents the limit of insufficient energy for droplet jetting, and the black dashed line represents the limit of onset of splashing [4,16,50]. (B) Comparison of the high-speed and long-distance printing capabilities. (C) Printing pattern demos.

Figure 6 compares the UHDV-PIJ method and the classical PIJ method in terms of the compatibility of ink materials and the capabilities of high-speed and long-distance printing, in order to verify the potential of the proposed UHDV-PIJ method.

Figure 6A compares the printable ink range of DOD inkjet printing methods (including PIJ and other inkjet methods), the classical PIJ method using the DMC11610 printhead, and the UHDV-PIJ method using the DMC11610 printhead in the parameter space of the Reynolds number *Re* and the Ohnesorge number *Oh*. A detailed description of *Re* and *Oh* for the ink used in the experiments can be found in Section S1. Printable inks used in DOD inkjet printing methods require appropriate fluid parameters of 0.1 ≤ *Oh* ≤ 1.0 (the green domain in the figure) [4,16,50]. As regards the classical PIJ method using the DMC11610 printhead, the appropriate fluid parameters for stable monodisperse droplet jetting recommended by the manufacturer are 0.36 ≤ *Oh* ≤ 0.72 and *Re* ≤ 18 (the domain enclosed by the brown line in the figure). As regards the UHDV-PIJ method using the DMC11610 printhead, monodisperse droplet jetting states are maintained even when *Oh* reaches 0.03 (viscosity of 1 cp and surface tension of 72.2 mN/m) and 1.18 (viscosity of 40.3 cp and surface tension of 64.1 mN/m), i.e., 0.03 ≤ *Oh* ≤ 1.18 (the green dashed lines in the figure), which is beyond the printable ink range provided by the classical PIJ method, and even beyond the printable ink range provided by the classical theory of DOD inkjet printing. Obviously, the UHDV-PIJ method proposed in this work can accommodate less optimal inks, allowing for a broader range of viscosity and surface tension to produce monodisperse droplets. In other words, the UHDV-PIJ method exhibits an ability to handle a wider range of inks.

Figure 6B gives an intuitive comparison for the capabilities of high-speed and long-distance printing between the classical PIJ and the UHDV-PIJ methods. In particular, experiments of printing patterns using different printheads under different printing speeds and printing distances were conducted (details in Section S2 and Figs. S6 to S8). From an overall perspective, it is clear that both the printing speed and printing distance have been greatly improved through the proposed UHDV-PIJ method. Table S3 provides statistics and comparisons of the improvements in the printing speed and printing distance in the above experiments. More specifically, under the optimal conditions of existing experimental data, the printing speed and printing distance were simultaneously increased by almost 10 times (printing speed increased by 12.5 times and printing distance increased by 9.21 times for the 9-μm nozzle printhead).

Figure 6C provides some printing linear pattern demos to demonstrate the UHDV-PIJ method’s ability of long-distance printing and “direct to shape/object” printing on irregular complex devices. The 9-μm nozzle printhead was selected, which has the same external dimensions as the 21-μm nozzle printhead. According to Table S3, the maximum printing distance can reach 6.5 mm at a printing speed of 2.5 m /s. The commercial ink BASE-CP12 (Shanghai Mifang Electronic Technology Co., Ltd.) is used, which appears black after curing at 150 °C for 20 min, increasing recognition. The droplet jetting process under the actuation waveform (0.1 μs–3.25 μs–0.1 μs–153.846 kHz, 15 V) designed by the UHDV-PIJ method is shown in Fig. S9. In Fig. 6C (i), the position relationship between the printhead and the substrate is displayed. The organic glass substrate has a height fluctuation of 5 mm on the surface, and the distance between the highest surface of the substrate and the printhead nozzle is 1 mm, which causes the printing distance to vary between 1 mm and 6 mm during the printing process. In Fig. 6C (ii to iv), the printed and solidified line patterns are conformally attached to complex surfaces such as zigzag surface, convex surface, and concave surface, with good shape. Figures S10, S11, and S12 show the overall morphology of the substrates before printing and after printing on zigzag surface, convex surface, and concave surface, respectively. The start and stop stages of the printing process are covered by shielding masks. All printed lines have a length of 50 to 60 mm in printing direction, and the overall morphology is very nice.

Table S4 summarizes the potential of the proposed UHDV-PIJ method compared to the classical PIJ method in terms of the (a) typical maximum droplet velocity, (b) typical maximum printing distance, (c) typical maximum printing speed, (d) droplet state at high velocity, and (e) compatibility of ink materials. Obviously, all key aspects exhibit obvious improvements, which constitutes a fundamental overall improvement.

## Discussion

To improve the droplet velocity of the PIJ technology, the mechanisms and actuation waveform design methods for improving droplet velocity through the positive crosstalk effect were systematically investigated in this work. By combining numerical and experimental results, the self-tuning mechanisms induced by the positive crosstalk effect were revealed, including self-reinforcing of the actuation pressure (increased by 2 to 4 times), self-restraining of satellite droplets (actively merged with subsequent main droplets), and self-weakening of the air resistance (reduced by 63%). These 3 tough and contradictory issues related to the improvement of the droplet velocity have been simultaneously addressed, achieving UHDV monodisperse droplet jetting. Based on the above mechanisms, a simulation-based waveform design method and an experimental-based waveform reverse design method were investigated, enabling the rapid waveform design for different inks and printheads. The results demonstrated that the UHDV monodisperse droplet jetting with a maximum velocity of 27.53 m/s can be achieved, reaching 3 to 5 times that of the classical PIJ method. Meanwhile, the compatibility of ink materials is expanded, since the Ohnesorge number and the viscosity of printable inks for the printhead used are increased from 0.36–0.72 to 0.03–1.18 and from 10–12 cp to 1–40.3 cp, respectively, even breaking the traditional limitations of the PIJ technology (Ohnesorge number of 0.1 to 1; viscosity of 1 to 25 cp). Furthermore, the printing speed and printing distance were increased by almost 10 times, demonstrating an ability of direct printing on zigzag surface, convex surface, and concave surface, which is a fundamental improvement that may promote the development of high-speed printing, long-distance printing, and “direct to shape/object” printing. Overall, this work provides a new perspective for improving droplet velocity and even offers a game-changing choice for expanding the boundaries of the PIJ technology.

## Materials and Methods

Details of experimental and numerical methods are presented in Sections S3 to S7, Figs. S13 to S19, and Tables S5 to S7.

## Data Availability

All data needed to evaluate the conclusions in the paper are presented in the paper and the Supplementary Materials. Additional data related to this paper may be requested from the authors.

## Supplementary Materials

Supplementary 1 Sections S1 to S7 Figs. S1 to S19 Tables S1 to S7 References
