# Supplementary material for: Piezoelectric Drop-on-Demand Inkjet Printing with Ultra-High Droplet Velocity
Source: Research (Wash D C). 2023 Oct 13;6:0248. doi: 10.34133/research.0248 (PMC10574180; doi:10.34133/research.0248)
Supplement: Supplementary 1 — Sections S1 to S7 Figs. S1 to S19 Tables S1 to S7 References [file research.0248.f1.docx]

# Supplementary Materials

Supplementary Materials for

**Piezoelectric drop-on-demand inkjet printing with ultra-high droplet velocity**

Zhengjie Yang^1^, Hongmiao Tian^1,*^, Chunhui Wang^1^, Xiangming Li^1,2^, Xiaoliang Chen^1,2^, Xiaoming Chen^1^, Jinyou Shao^1,2,*^

^1^ State Key Laboratory for Manufacturing Systems Engineering, Xi’an Jiaotong University, Xi’an, Shaanxi 710049, China.

^2^ Frontier Institute of Science and Technology (FIST), Xi’an Jiaotong University, Xi’an, Shaanxi 710049, China.

^*^ Address correspondence to: [hmtian@xjtu.edu.cn](mailto:hmtian@xjtu.edu.cn) (H.T.); [jyshao@xjtu.edu.cn](mailto:jyshao@xjtu.edu.cn) (J.S.)

The file includes:

Sections S1 to S7

Figs. S1 to S19

Tables S1 to S7

Supplementary References:

**Supplementary Notes:**

## *Section S1. Details of Re and Oh for the ink used in the experiments*

The Reynolds number Re mainly represents droplet velocity and the Ohnesorge number Oh mainly represents materials properties of viscosity and surface tension.

As it is well known, printable inks used in all DOD inkjet printing methods require appropriate fluid parameters, which need to have intermediate values in the parameter space composed of *Re* and *Oh* (green domain). And inks are too viscous to jet under the condition of Oh>1 (the blue domain), while satellite droplets can not be controlled under the condition of Oh<0.1 (the orange domain) [R1, R2, R3].

As regards the classical PIJ method using the DMC11610 printhead, the appropriate fluid parameters for stable monodisperse droplet jetting recommended by the manufacturer are a viscosity of 10-12 cp and a surface tension of 28-42 mN/m, corresponding to Oh value of 0.36-0.72 (the brown line in Fig. 6 a), in which the density of the ink is selected in the normal range of 1000 kg/m^3^ to 1200 kg/m^3^, and the radius of the droplet is selected in the normal range of 10 μm to 15 μm. The maximum Re obtained is ~18, in which the droplet velocity is selected as 10 m/s, corresponding to the maximum droplet velocity could be obtained in the classical PIJ method. Thus, printable inks used for the classical PIJ method using the DMC11610 printhead require the parameter space of 0.36 ≤ Oh ≤ 0.72 and Re ≤ 18 (the domain enclosed by the brown line in Fig. 6 a).

## *Section S2. Details of the experiments of printing patterns using different printheads under different printing speeds and printing distances*

The most basic but representative dot patterns were printed in the experiments, and the distribution of the dot landing positions is used to determine whether the corresponding printing capability under the selected parameters has been achieved. In general, the the landing positions of the printed dots are significantly affected by the airflow produced by inappropriate printing speeds and printing distances, causing irregular printed patterns (Fig. S6). Experimental photographs of patterns printed with different printheads under different printing speeds and printing distances are shown in Figs. S7 and S8.

Fig. S7 displays the experimental photographs for the printhead with a nozzle diameter of 9 μm (DMC-11601). In Figs. S7 a-(i-iii), the experimental photographs obtained in the classical PIJ experiments (waveform of 0.1 μs - 3.3 μs - 0.1 μs - 1 kHz and actuation voltage of 15 V) are displayed. For a printing speed of 0.05 m/s, the printed dots with a size of 11-12 μm (on a hydrophobic PET substrate) are evenly distributed along the printing direction under the printing distances of 1 mm, 1.25 mm, and 1.5 mm, meeting the requirements for high-resolution printing. When the printing distance is increased to 1.75 mm and 2 mm, significant deviations in the landing positions of the printed dots can be observed. For certain ordinary applications with low precision requirements, e.g., coatings, such printing distances can be used to a certain extent. This indicates that the maximum printing distances is 1.5 mm under the printing speed of 0.05 m/s. Correspondingly, the maximum printing distances are 1.25 mm and 0.7 mm under the printing speeds of 0.1 m/s and 0.2 m/s, respectively. For higher printing speeds and larger printing distances (Figs. S7 b, c, and d), the landing positions of the printed dots are completely irregular, almost without any practical value. In Fig. S7 a-iv, the experimental photographs obtained in the UHDV-PIJ experiments (waveform of 0.1 μs - 3.3 μs - 0.1 μs - 151.515 kHz and actuation voltage of 15 V) are displayed. For a printing speed of 2.5 m/s, the printed dots with a size of 15-16 μm are evenly distributed along the printing direction under the printing distances of 5 mm, 5.25 mm, 5.5 mm, 5.75 mm, 6 mm, 6.25 mm, and 6.5 mm. The maximum printing distance is 6.5 mm under the printing speed of 2.5 m/s. These results suggest that the proposed the UHDV-PIJ method can simultaneously improve both the printing speed and printing distance. For example, when the printing speed is increased by 50 times, the printing distance increases by 4.33 times (Figs. S7 a-iv vs. a-i); when the printing speed is increased by 25 times, the printing distance increases by 5.20 times (Figs. S7 a-iv vs. a-ii); when the printing speed is increased by 12.5 times, the printing distance increases by 9.29 times (Figs. S7 a-iv vs. a-iii).

Fig. S8 displays the experimental photographs for the printhead with a nozzle diameter of 21 μm (DMC-11610). In Fig. S8 a, the experimental photographs obtained in the classical PIJ experiments (waveform of 0.1 μs - 3.55 μs - 0.1 μs - 1 kHz and actuation voltage of 25.5 V) are displayed. The size of the printed dots is about 47-50 μm (on a hydrophilic silica substrate). The maximum printing distances are 4 mm, 3 mm, 2.5 mm, and 2 mm under printing speeds of 0.05 m/s, 0.1 m/s, 0.2 m/s and 0.25 m/s, respectively. In Fig. S8 b, the experimental photographs obtained in the UHDV-PIJ experiments (waveform of 0.1 μs - 3.55 μs - 0.1 μs - 142.857 kHz and actuation voltage of 25.5 V) are displayed. For a printing speed of 2.5 m/s, the printed lines with a width of 80-85 μm are evenly distributed along the printing direction under the printing distances of 6-17 mm. The maximum printing distance is 17 mm under the printing speed of 2.5 m/s. These results indicate that the proposed the UHDV-PIJ method can simultaneously improve both the printing speed and printing distance. For instance, when the printing speed is increased by 50 times, the printing distance increases by 4.25 times (Figs. S8 b vs. a-i); when the printing speed is increased by 25 times, the printing distance increases by 5.67 times (Figs. S8 b vs. a-ii); when the printing speed is increased by 12.5 times, the printing distance increases by 6.8 times (Figs. S8 b vs. a-iii); when the printing speed is increased by 10 times, the printing distance increases by 8.5 times (Figs. S8 b vs. a-iv).

It can be observed that there is a contradiction between the printing speed and the printing distance. Reducing the printing speed can improve the printing distance, correspondingly reducing the printing distance can improve the printing speed. Obviously, the printing distances of 6.5 mm and 17 mm are far from reaching the maximum distance, i.e., throwing distance, where droplet velocity decreases to zero, for the 9 μm and 21 μm nozzle printheads, respectively. In general, compared to the classical PIJ method, the proposed UHDV-PIJ method can significantly improve printing speed and printing distance at the same time.

The smaller the droplet, the more significant the deceleration effect caused by the air drag, resulting in a smaller droplet flying distance. This is the reason why the flying distance of small droplet (9 μm nozzle) is much smaller than that of large droplet (21 μm nozzle) under the same droplet velocity and printing speed. The distance between the printhead and the substrate becomes very small as the droplet size <10 μm, which probably limit the practical small nozzle size for piezoelectric inkjet printing [R4]. A practical example is that the printing distance at a general printing speed of 0.5 m/s for a 9 μm nozzle printhead could be too small to measure, and the maximum printing distance could only reach 0.7 mm even if the printing speed is reduced to 0.2 m/s (Table S3). Besides, the material deposition rate is probably another limitation of the practical small nozzle size for piezoelectric inkjet printing. At the same jetting frequency, the material deposition rate is proportional to the droplet volume; that is, the material deposition rate is proportional to cube of nozzle diameter. At the traditional low jetting frequency (< 20 kHz for the printhead used), a very low material deposition rate could be achieved for a small nozzle size printhead. Therefore, the proportion of commercial printhead with a nozzle diameter of <10 μm in the entire market is currently very small.

The strategy proposed in this work achieves droplet jetting at a high droplet velocity of 26.7m/s and a high jetting frequency of 151.515 kHz for the 9 μm nozzle printhead, which means that the printing distance and material deposition rate that restrict the use of small nozzle printhead are simultaneously improved. From this perspective, this work enables the application of small nozzle printhead in a wider range of printing scenarios, which may indeed provide a direction for promoting the development and commercialization of small nozzle printhead.

## *Section S3. Details of the experimental apparatus*

The experimental apparatus (Figs. S13 and S14) incorporated three modules, i.e., droplet generation, droplet observation and droplet printing. The droplet generation module drives and controls the PIJ printhead to achieve droplet jetting; the droplet observation module captures droplet images; the droplet printing module implements pattern printing on the substrate.

Droplet generation module comprised a piezoelectric printhead, back pressure unit and actuation unit (including Keysight 33512b arbitrary waveform generator and TEGAM-2350 power amplifer). Back pressure unit provided a positive pressure of 0.05 to 0.1 MPa for cleaning the printhead and a negative pressure of -5 kPa for improving droplet jetting stability. Actuation unit could produce actuation waveforms with an amplitude ranging from -50 to 50 V, and the minimum edge time was less than 100ns.

Droplet observation module adopted the Jetxpert stroboscopic droplet observation system (Imagexpert, Inc.), in which the exposure time was set to 500ns and the droplet images were captured with the image pixel size of 1.223 μm or 2.587 μm corresponding to the magnification of roughly 3X and 1.5X, respectively.

Droplet printing module had a belt conveyor driven by the brushless motor, and the substrate was fixed on the conveyor. The printing speed V_p_, which could reach 0.05 to 2.5 m/s, was setted by adjusting the motor speed. The printing distance, which could reach 0 to 50 mm, was setted by changing the conveyor height and the screw lift (displacement accuracy:10 μm).

For the PIJ printhead used (DMC-11610 printhead and DMC-11601 printhead), the recommended inks should have the viscosity of 10-12 cp and the surface tension of 28-42 mN/m at jetting temperature. And the inks should be filtered with a 0.2 µm filter and degassed by vacuum. In addition, the inks should also be rested for nearly 24 h before using. [R5]

The experiment was performed in a clean room at 25 ℃ (± 2 ℃).

## *Section S4. Details of the numerical method*

A numerical model (Fig. S15) consisting of an actuation simulation unit and a droplet jetting simulation unit was developed to simulate the physical phenomena taking place inside and outside the printhead during the droplet jetting process. All simulations were performed in the COMSOL Multiphysics software.

In **actuation simulation unit**, the flow rule of the ink inside the printhead controlled by the actuation waveform will be analyzed, aiming to solve the issue of the actuation pressure:

Deformation of the piezoelectric actuator under the control of the actuation waveform was modeled by the piezoelectric coupling equations:

$S=\text{s}_{E}T+d^{T}E$ (S1)

$D=dT+\varepsilon_{0}\varepsilon_{rT}E$ (S2)

where ***S*** is the strain, s_E_ is the stiffness constants, ***T*** is the stress, *d* is the piezoelectric coupling constants, ***E*** is the electric field, ***D*** is the electric flux density, ε_rT_ is the relative dielectric constant, and ε_0_ is the vacuum dielectric constant.

The laminar flow (including ink flow and air flow) is governed by the incompressible Navier Stokes equations:

$\rho\frac{\partial u}{\partial\text{t}}-\nabla\cdot[-pI+\mu(\nabla u+(\nabla u)^{T})]+\rho(u\cdot\nabla)u=F_{st}+\rho g$ (S3)

$-\nabla\cdot u=0$ (S4)

where *ρ* is the fluid density, *μ* is the dynamic viscosity, ***u*** is the velocity, *p* is the pressure, ***I*** is the unit diagonal matrix, ***F_st_*** is the surface tension on the ink/air interface, and ***g*** is the gravity. The meniscus motion at nozzle was captured by a moving mesh method [R6].

The bidirectional interaction between the solid structure of the printhead and the ink was modeled by the fluid-solid coupling equations:

$F_{T}=-n\cdot(-pI+\mu(\nabla u+(\nabla u)^{T}))$ (S5)

where ***n*** is the normal vector of the boundary and ***F****_T_* is the sum of the pressure and viscous forces.

The boundary conditions and the mesh division were shown in Fig. S16. The total number of meshes was set to 1395232 (minimum mesh size 1 μm) in the simulation to maintain mesh convergence, and the meshes near the deformed interface were remeshed every 0.05 μs. The maximum time step size was limited to 0.05 μs.

In **droplet jetting simulation unit**, the law of the droplet jetting at the nozzle and the subsequent free flying process will be investigated, aiming to solve the issues of satellite droplets and the air resistance:

The droplet jetting simulation model is a two-dimensional axi-symmetric model. The laminar flow of droplets and air outside the printhead was also modeled by the incompressible Navier-Stokes equations (Eqs. (S3) and (S4)).

The ink/air interface motion was captured by a phase-field method [R7], in which the two-phase dynamics is governed by Cahn-Hilliard equations:

 (S6)

 (S7)

where *ϕ* is the dimensionless phase filed variable, ***u*** is the velocity, *γ* is the mobility, *λ* is the mixing energy density, *ε* is the interface thickness parameter, and *ψ* is the phase field help variable. The liquid/air interface is defined as the region where the dimensionless phase field variable *ϕ* goes from -1 to 1, and the volume fractions of the ink (*V_ink_*) and air (*V_air_*) can be calculated by:

 (S8)

 (S9)

The fluid density *ρ* and the viscosity *μ* of the interface region can be calculated by:

$\rho=\rho_{ink}+(\rho_{air}-\rho_{ink})V_{air}$ (S10)

$\mu=\mu_{ink}+(\mu_{air}-\mu_{ink})V_{air}$ (S11)

where *ρ_ink_* and *ρ_air_* are the density of ink and air respectively, *μ_ink_* and *μ_air_* are the viscosity of ink and air respectively.

The ***F_st_*** in equation (3) can be computed by:

 (S12)

where ***G*** is the chemical potential, which can be computed by:

$G=\frac{\lambda}{\varepsilon^{2}}\psi$ (S13)

The *λ* and *ε* in equation (6) are related to the surface tension coefficient of the ink/air interface by equations below:

$\sigma=\frac{2\sqrt{2}}{3}\frac{\lambda}{\varepsilon}$ (S14)

The boundary conditions and the mesh division were shown in Fig. S17. The time-adaptive mesh refinement method was used to remesh the model at distinct time intervals (10 μs), in which the regions requiring fine meshes would move over time to obtain accurate calculation results in a reasonable solution time with sufficiently fine meshes. The minimum mesh size reaches 0.5 μm. The maximum time step size was limited to 0.05 μs. The pressure curve at the nozzle inlet extracted from the actuation simulation unit was used as the the input condition of the droplet jetting simulation unit, in which way the internal and external simulation of the printhead were connected. It should be noted that only a small portion of the experimental actuation voltage was applied in the actuation simulation unit to improve the calculating efficiency, thus the extracted pressure value at the nozzle inlet should be magnified before use.

## *Section S5. Details of method for characterizing droplet velocity*

Droplet velocity: so easy to state, routine to measure, a lot harder to explain and far harder still to use [R8]. The average droplet velocity could be obtained by dividing the droplet travel distance by the total travel time. Apart from that, this article also focuses on the instantaneous droplet velocity. Droplet velocity (instantaneous droplet velocity) changes dynamically during droplet forming and flying processes. The typical droplet velocity variation characteristics and droplet jetting process were illustrated in Fig. S18. According to time sequences, droplet velocity would go through three stages after the printhead was fired. First, ink would be rapidly pushed out of the nozzle, tip speed of the jet-flow increased from zero to the maximum value (V_tip_). Second, the speed of the jet-flow would decrease quikly from the maximum value, since the ink inside the nozzle would flow inwards under the reverse actuation pressure, in the mean time the ink ouside the nozzle continued to flow outwards under the inertia force. The jet flow broke up to form monodisperse droplets, at which time the droplet velocity was defined as the initial droplet velocity V_0_. In general, V_0_ is only roughly half the size of V_tip_. Finally, droplet velocity would gradually decrease under the action of the air resistance until droplet hitted the substrate. At this stage, droplet velocity decreased slowly with the increase of the distance to the nozzle. Droplet velocity was characterized as V_D_ at the position where the distance from the nozzle is D.

In actuanl application, attentions should be paid to the droplet velocity in the third stage, since the setting of the printing distance makes the droplet hitting the substrate in this stage. Therefore, in this study, it is generally necessary to add the corresponding distance from the droplet to the nozzle as a reference to characterize droplet velocity. For convenience, we abbreviate it as V_D_ @ D, e.g. droplet velocity of 5 m/s at the position where the distance from the droplet to the nozzle is 1 mm would be abbreviated as 5 m/s @ 1 mm. Droplet velocity, which equals to the travel distance divided by the travel time, was measured by the ImageJ software. In the measurement, it was assumed that t_D_ is the time when droplet reach the position where the distance from the droplet to the nozzle equals to D. Correspondingly, t_1_ and t_2_ are equal to t_D_ - 0.5 μs and t_D_ + 0.5 μs, D_1_ and D_2_ are the distance between the droplet and the nozzle at the time of t_1_ and t_2_, respectively. The formula for calculating drop velocity can be expressed as:

$V_{D}=\frac{D_{2}\text{-}D_{1}}{t_{2}-t_{1}}$ (S15)

## *Section S6. Details of the physical properties of the inks*

Inks with six different viscosities were used, including 1.0 cp, 5.1 cp, 9.2 cp, 12.5 cp, 17.9 cp, 25.2 cp and 40.3 cp, which had different mass ratios of glycerine and water. As it can be seen, the minimum and maximum viscosity were 1.0 cp and 40.3 cp, respectively, covering the viscosity range of common inks used in the PIJ technology (1-25 cp). The physical properties of the glycerine-water inks are listed in Table S5. The viscosity and surface tension of the inks were measured under ambient temperature (25 ℃), and the ink density was calculated by dividing the mass by the volume.

## *Section S7. Details of the printheads used*

Common commercial PIJ printheads with a nozzle diameter of 21 μm and 9 μm (DMC-11610 and DMC-11601, Fujiflm Dimatix, Inc., respectively) were selected as representative examples for the corresponding simulation and experimental studies. As regards the DMC-11610 printhead, its structural parameters were experimentally measured and the materials parameters were estimated based on the conventional materials parameters; the peripheral length of the nozzle surface of the printhead is 54mm, and the peripheral width of the nozzle surface of the printhead is 14mm; further details are introduced in Fig. S19 and Tables S6 and S7. It should be noted that in the selection of the printheads, the convenience of the existing experimental conditions was mainly considered. At the same time, the selected printheads are quite cheap and have been widely used for nearly 20 years, which is beneficial for other researchers to verify the methods proposed in this article. Moreover, the proposed method can also be used for any other commercial PIJ printheads.

## *Fig. S1.*

**
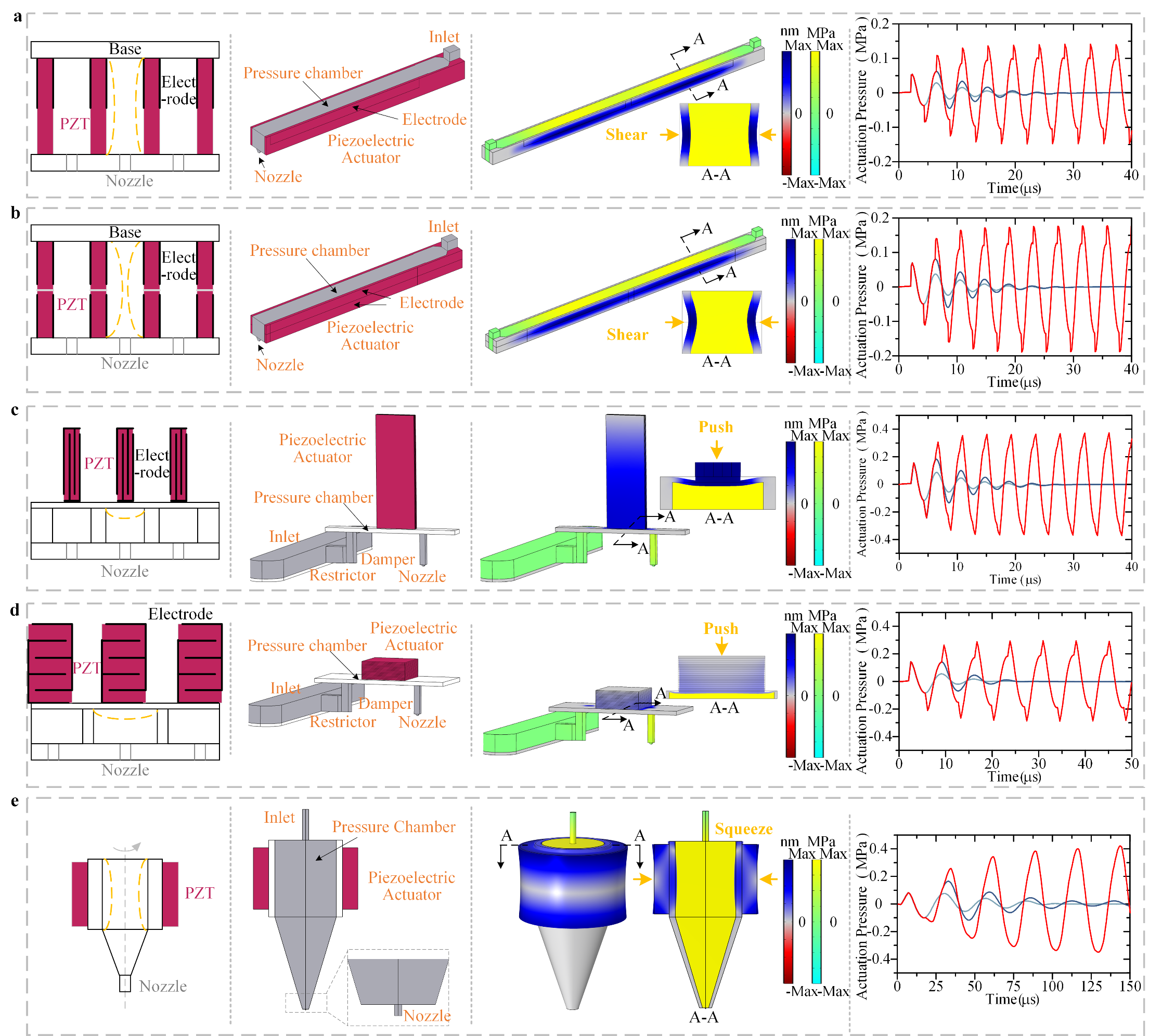
**

**Fig. S1.** Simulation results of the actuation pressures of different printhead structural forms: (a) and (b) shear mode, such as XAAR 1003 and XAAR 128 printheads; (c) push mode, such as EPSON MACH technology; (d) push mode, such as RICOH Gen5s technology; (e) squeeze mode, such as Microfab technology.

## *Fig. S2.*


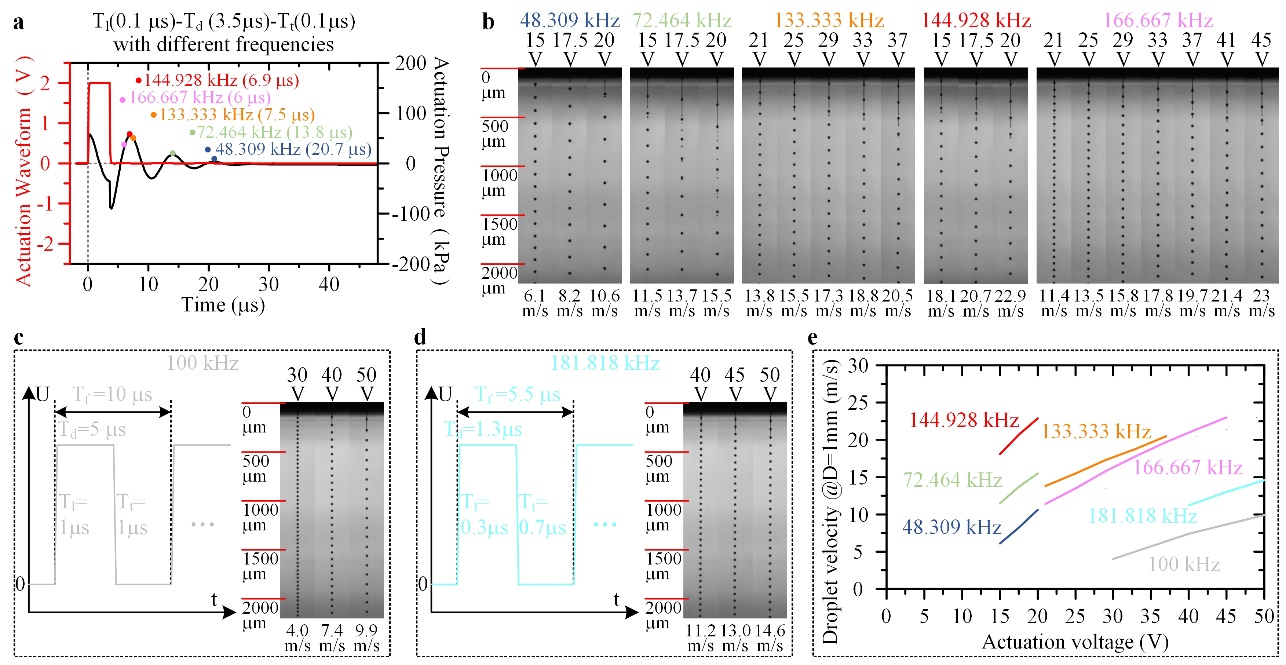


**Fig. S2.** (a) The different superimposing position under different frequencies under the waveform of 0.1 μs-3.5 μs-0.1 μs; (b) The droplet jetting state with different actuation voltage under different frequencies; (c) The droplet jetting state at 100 kHz under the waveform of 1 μs-5 μs-1 μs; (d) The droplet jetting state at 181.818 kHz (5.5 μ s) under the waveform of 0.3 μs-1.3 μs-0.7 μs; (e) The variation of droplet velocity with increasing actuation voltage at different frequencies.

## *Fig. S3.*

**
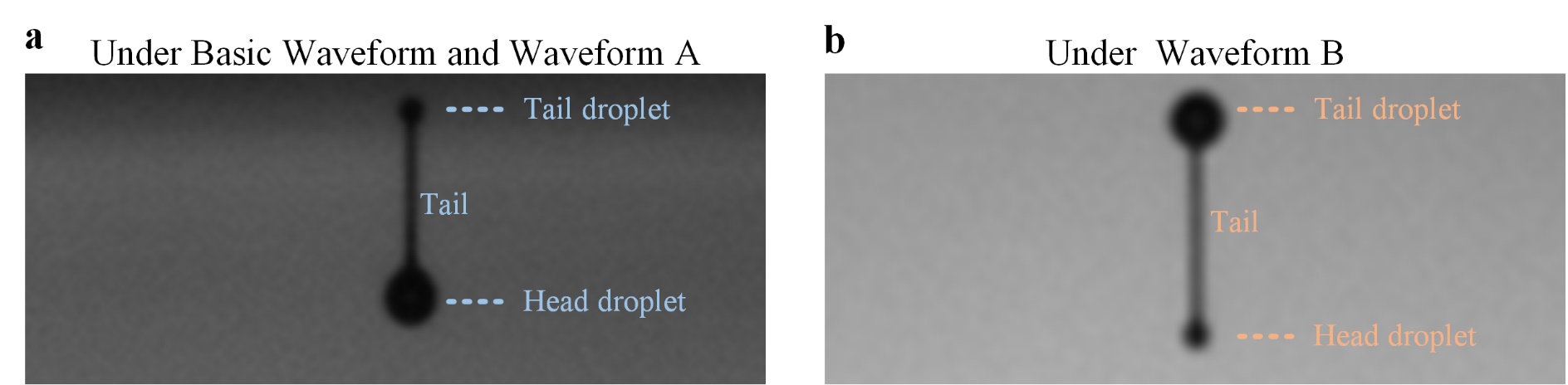
**

**Fig. S3.** The morphologies of formed droplets under low and high frequency.

## *Fig. S4.*


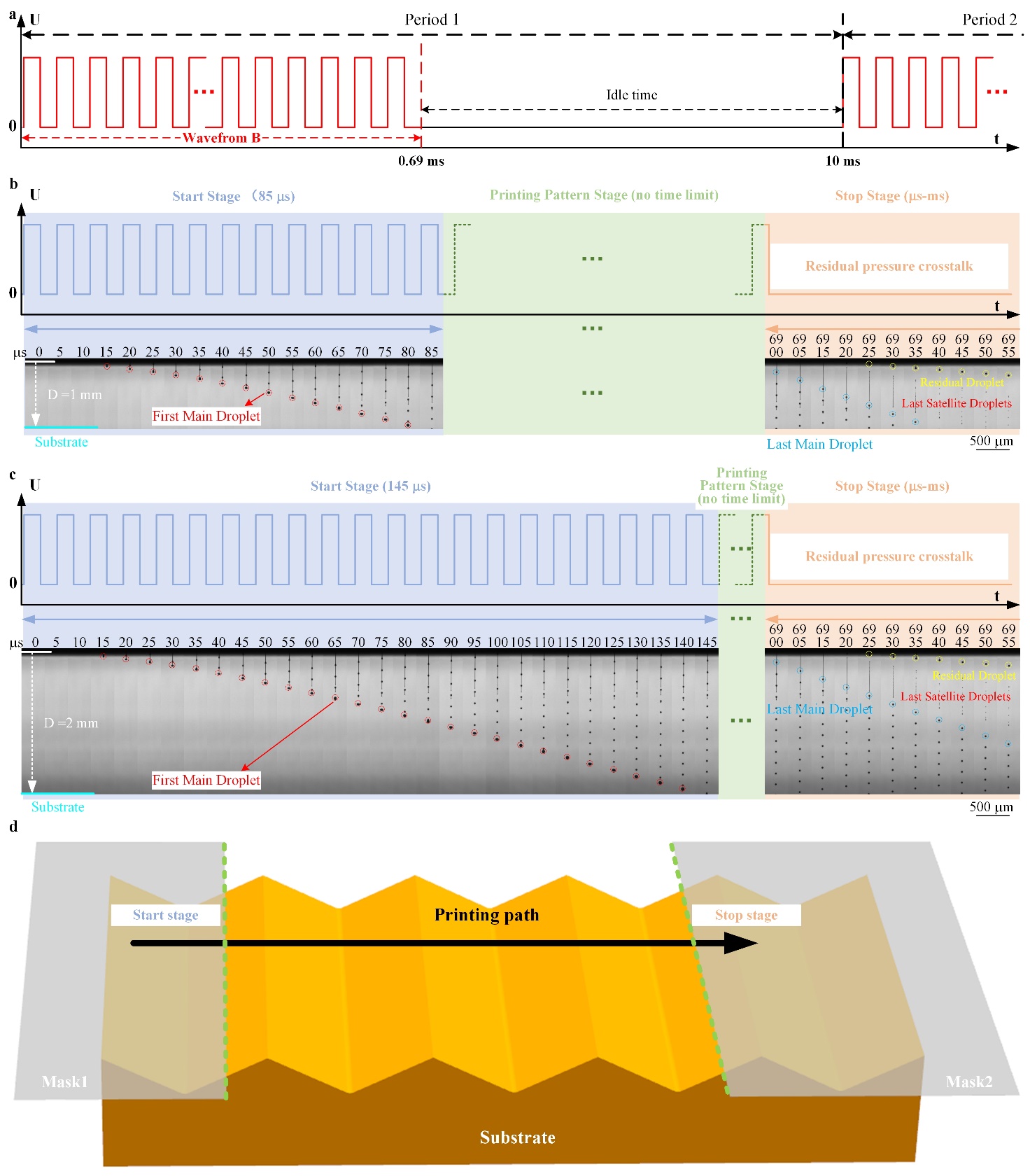


**Fig. S4.** Evaluation of the start and stop processes.

## *Fig. S5.*

**
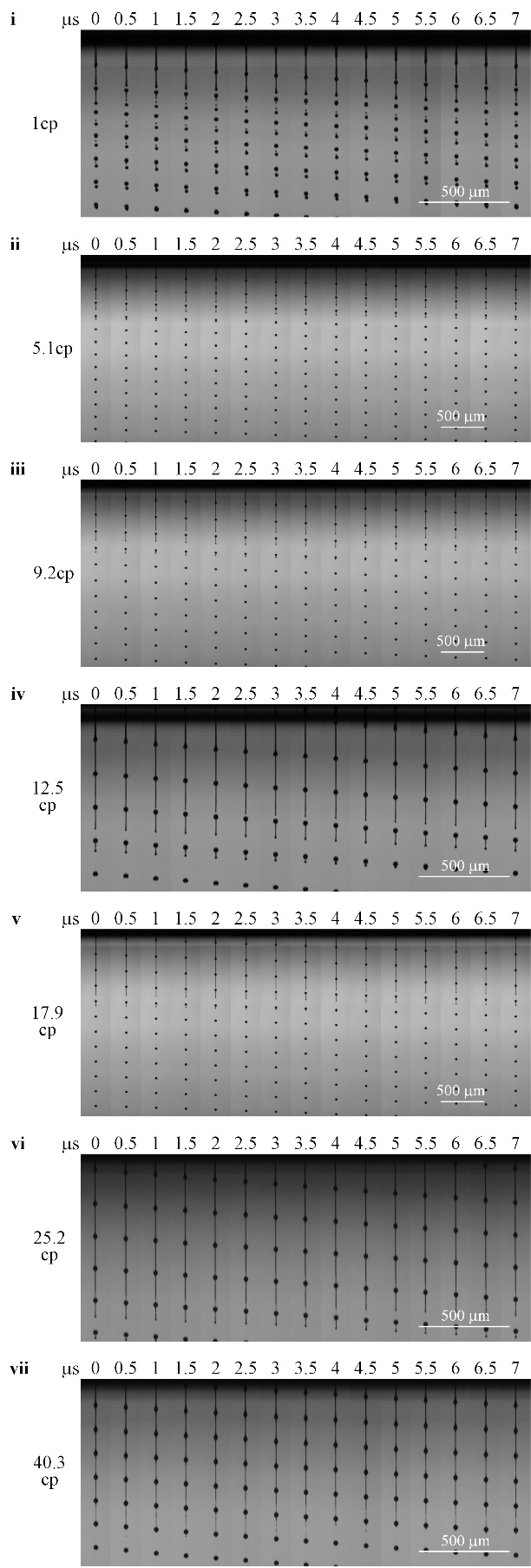
**

**Fig. S5.** The detailed droplet jetting processes at the maximum actuation voltage for different inks.

## *Fig. S6.*

**
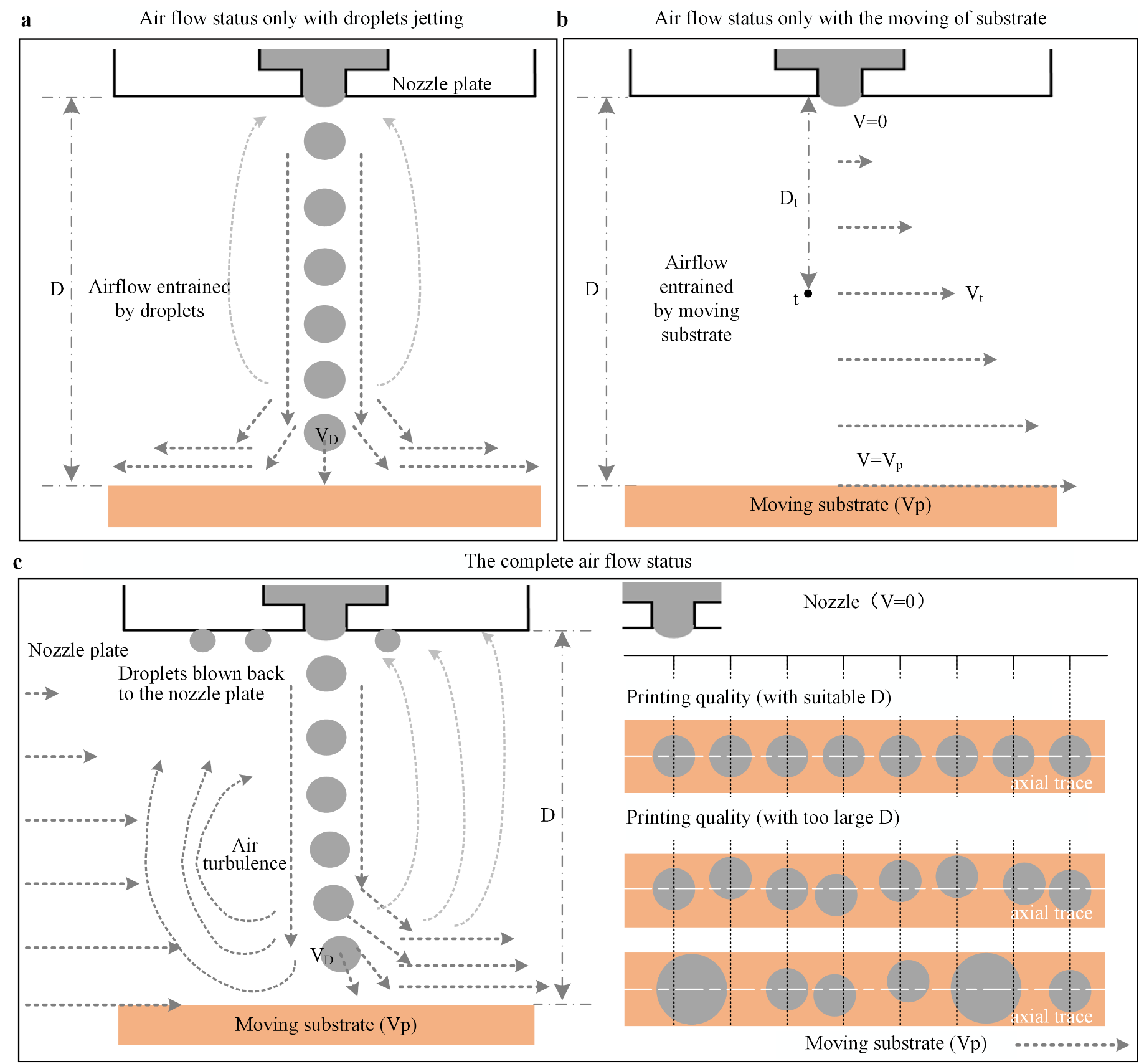
**

**Fig. S6.** Air flow during inkjet printing process and it’s influence on printing quality.

## *Fig. S7.*

**
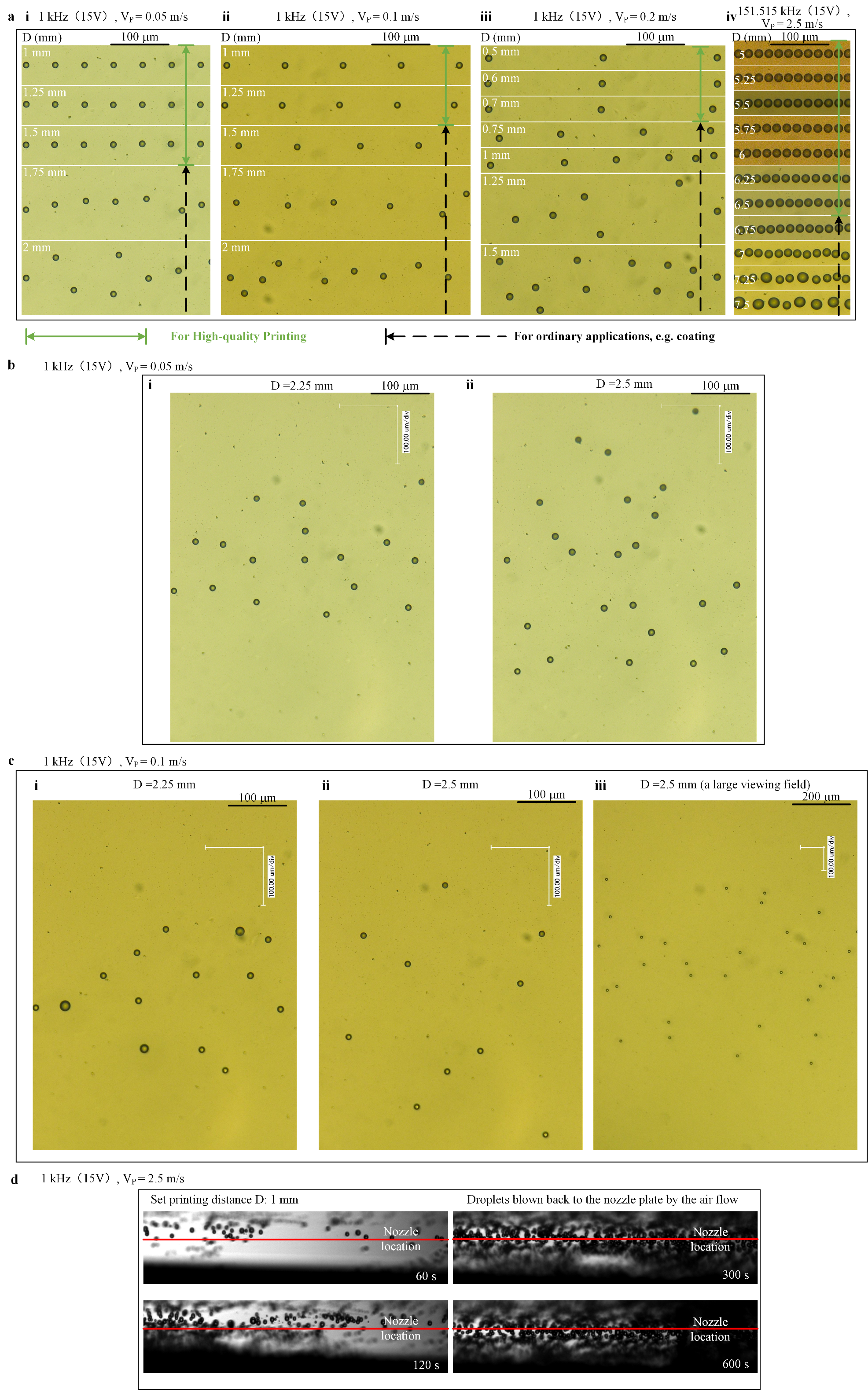
**

**Fig. S7.** Experimental photographs of printed patterns obtained with the printhead with a nozzle diameter of 9 μm.

## *Fig. S8.*


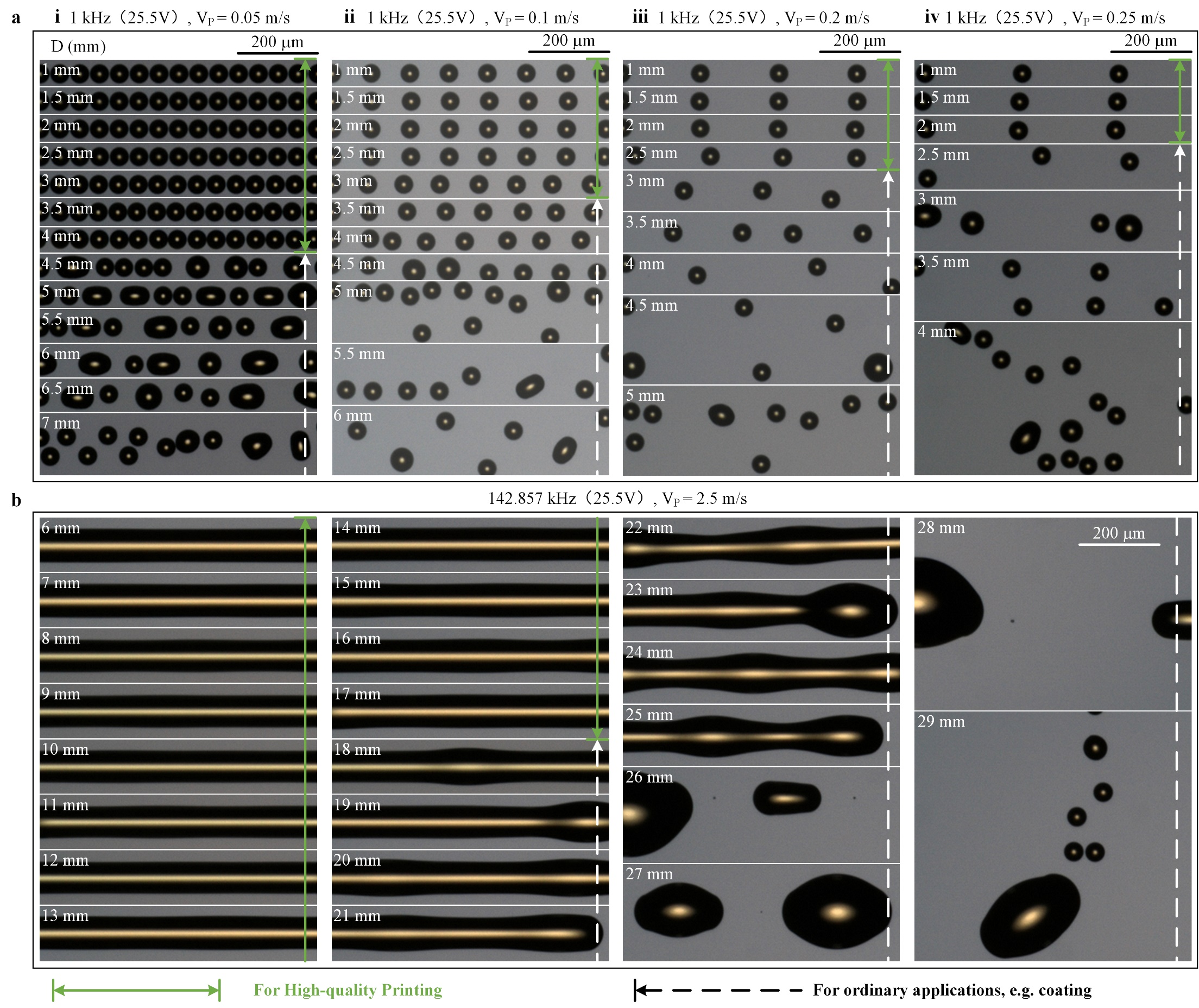


**Fig. S8.** Experimental photographs of printed patterns obtained with the printhead with a nozzle diameter of 21 μm.

## *Fig. S9.*


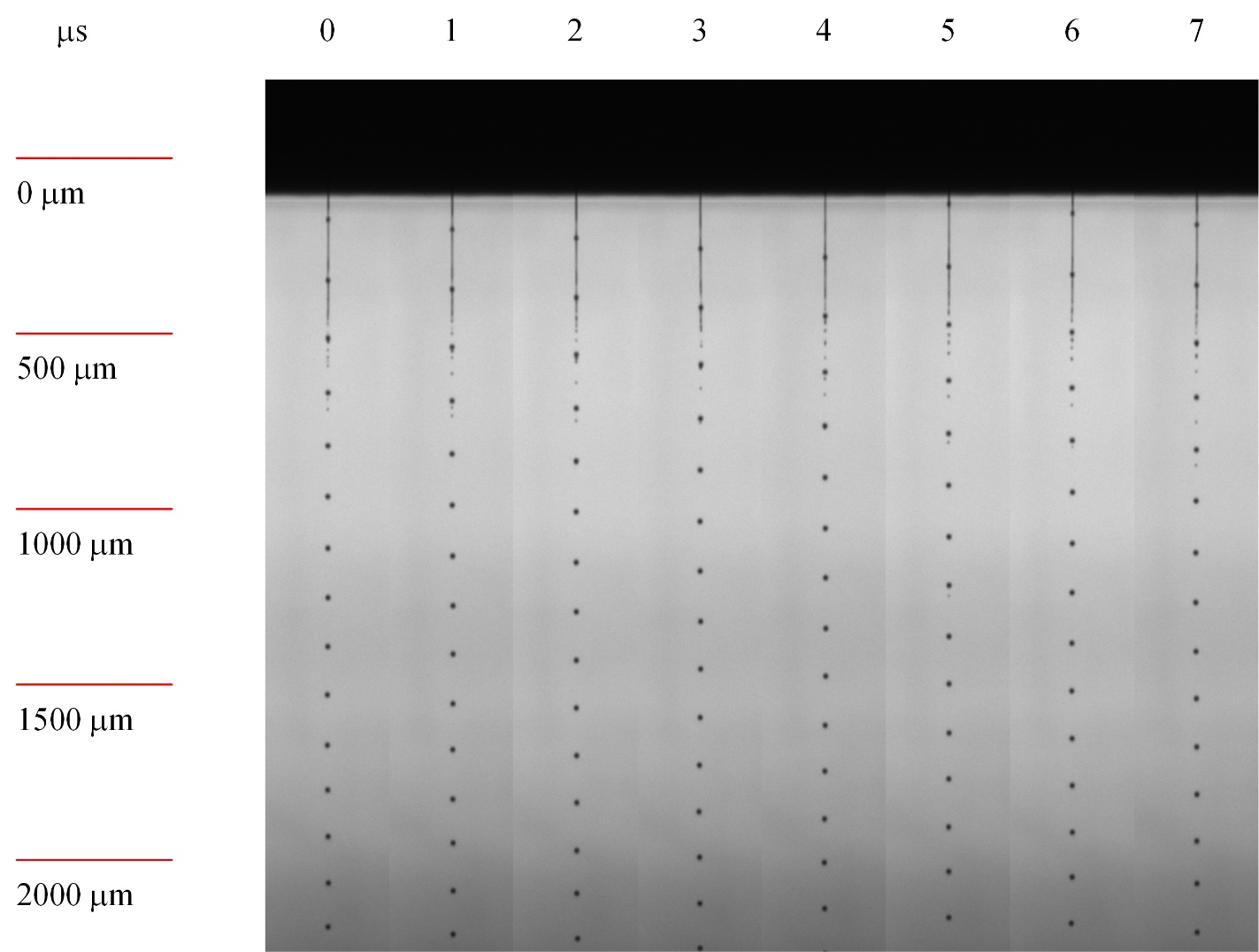


**Fig. S9.** The droplet jetting process under the actuation waveform of 0.1 μs-3.25 μs-0.1 μs-153.846 kHz (15V).

## *Fig. S10.*


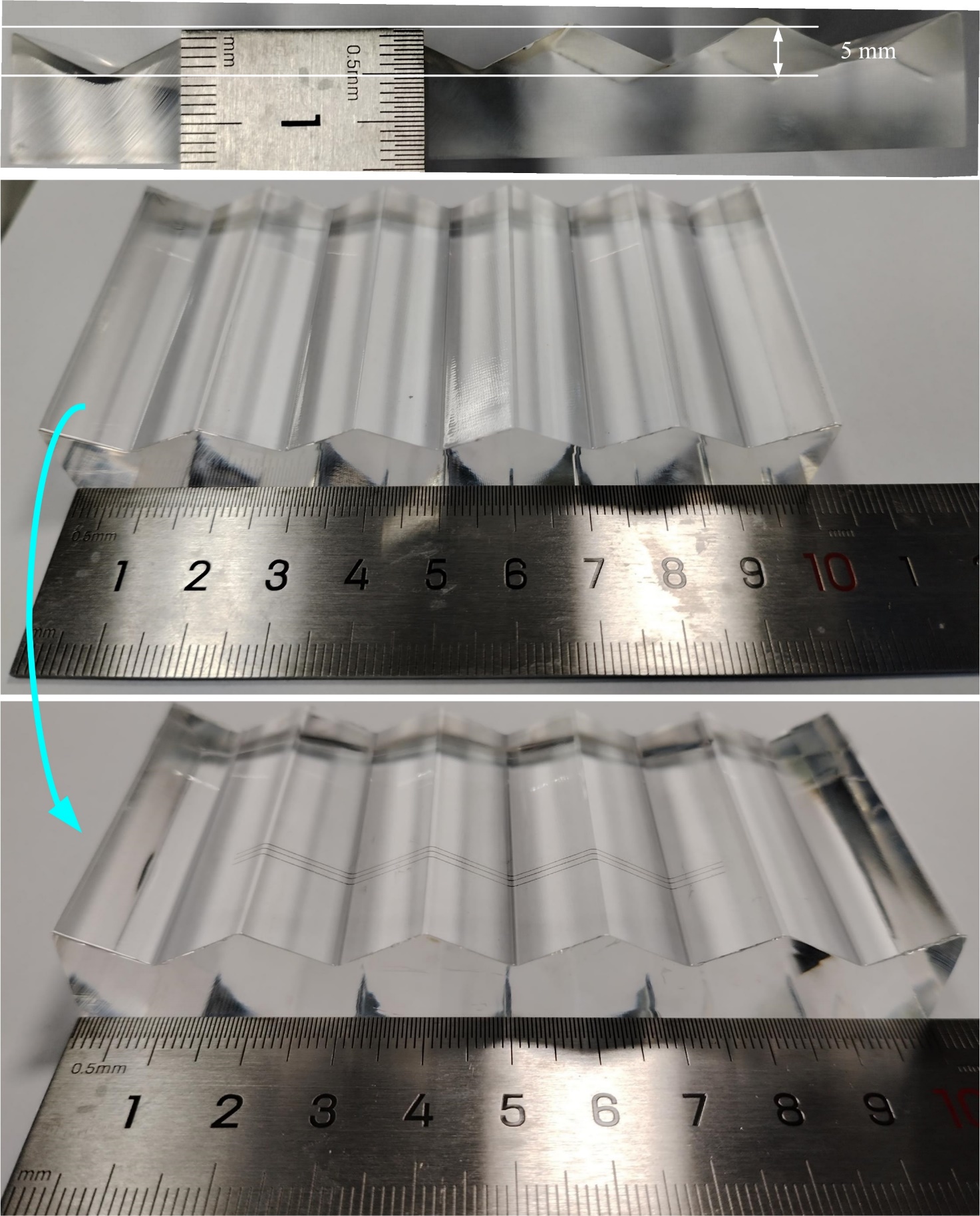


**Fig. S10.** The overall morphology of the substrates before printing and after printing on zigzag surface.

## *Fig. S11.*


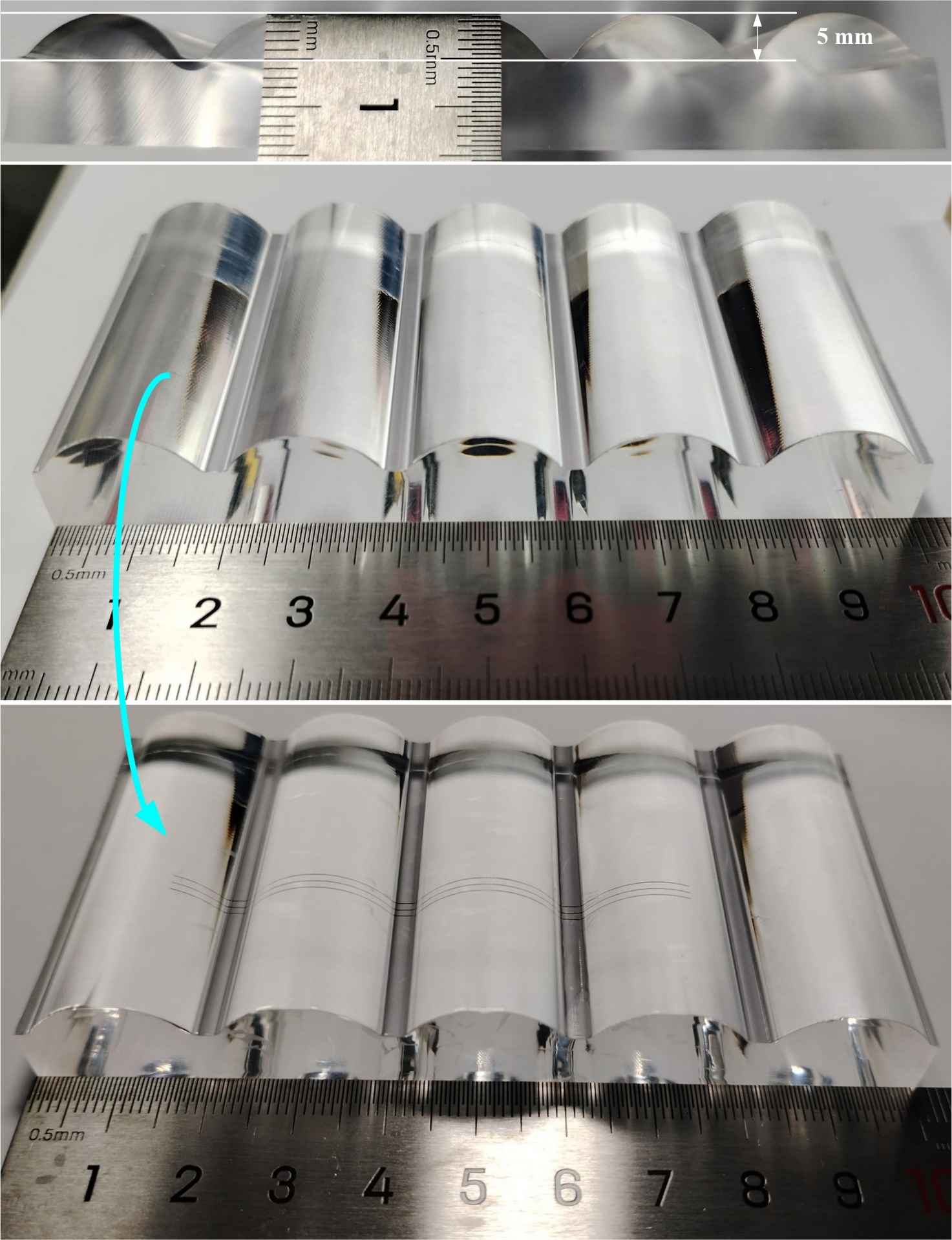


**Fig. S11.** The overall morphology of the substrates before printing and after printing on convex surface.

## *Fig. S12.*


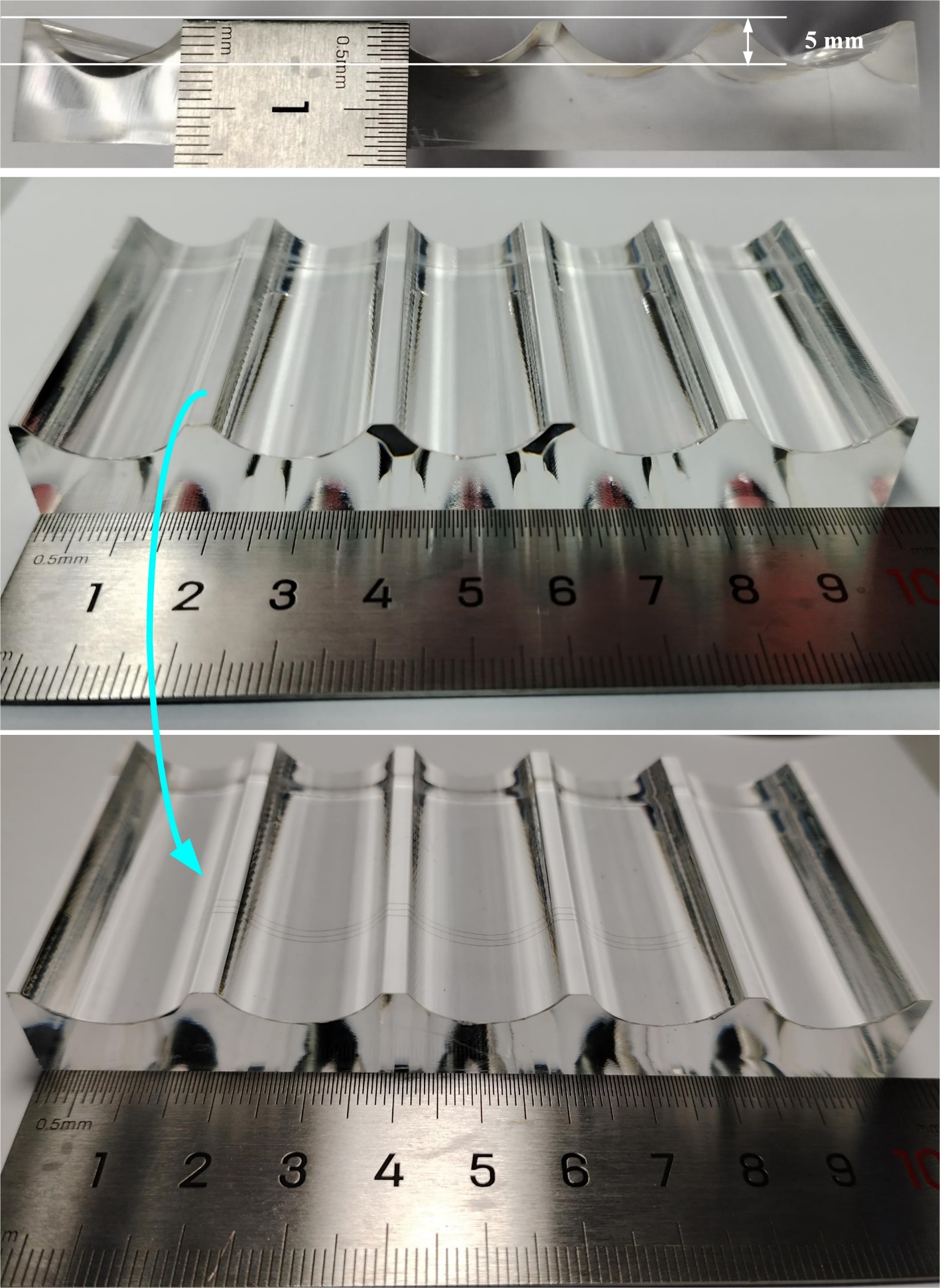


**Fig. S12.** The overall morphology of the substrates before printing and after printing on concave surface.

## *Fig. S13*


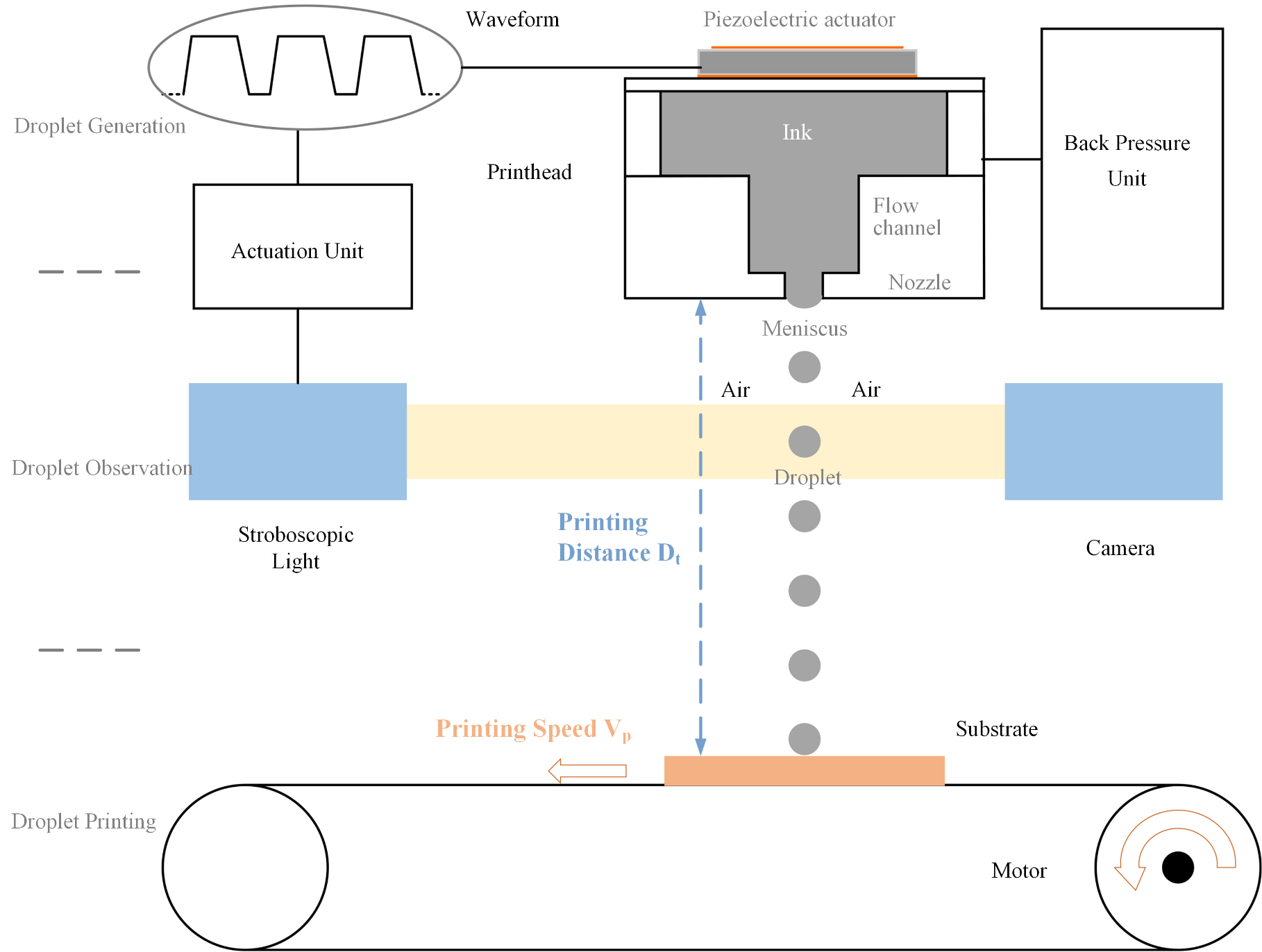


**Fig. S13**. Schematic of the experimental apparatus.

## *Fig. S14*

**
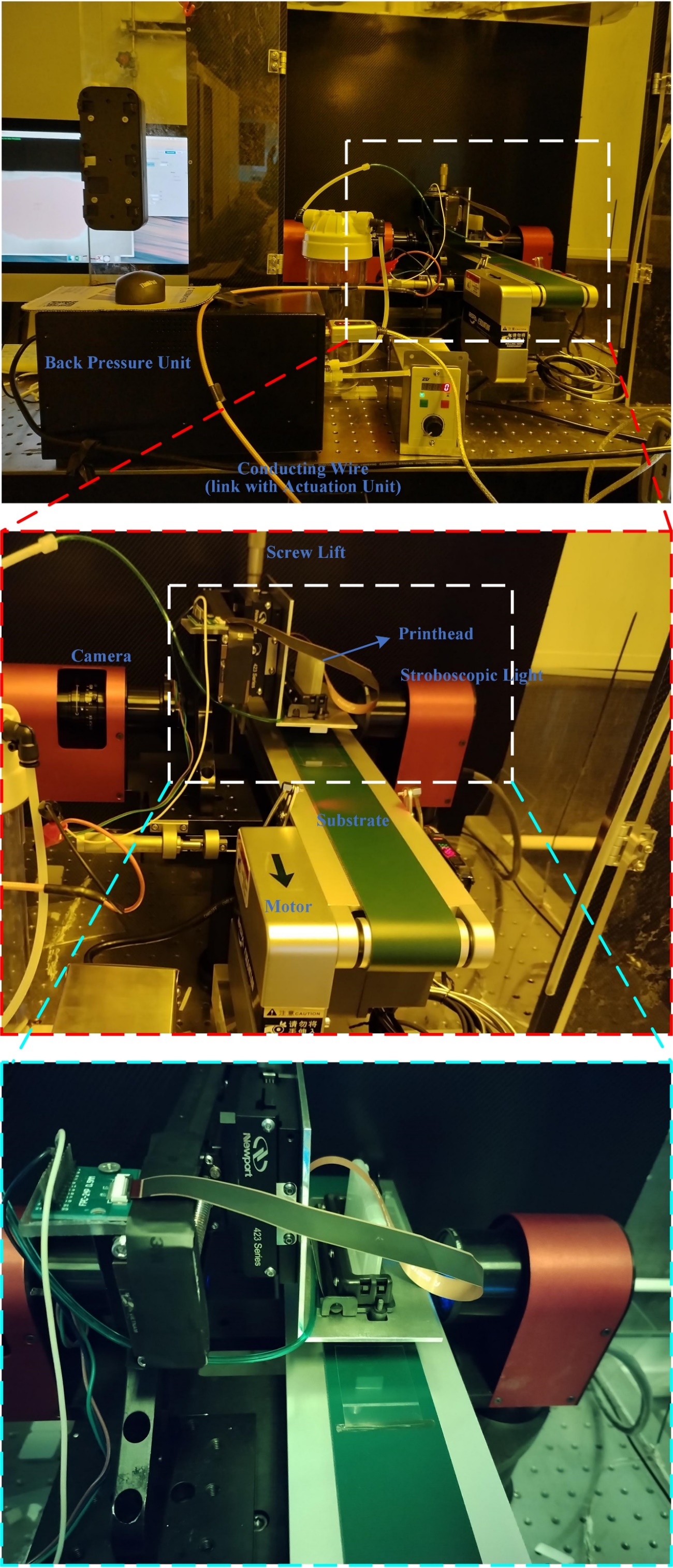
**

**Fig. S14** Photographs of the experimental devices.

## *Fig. S15.*


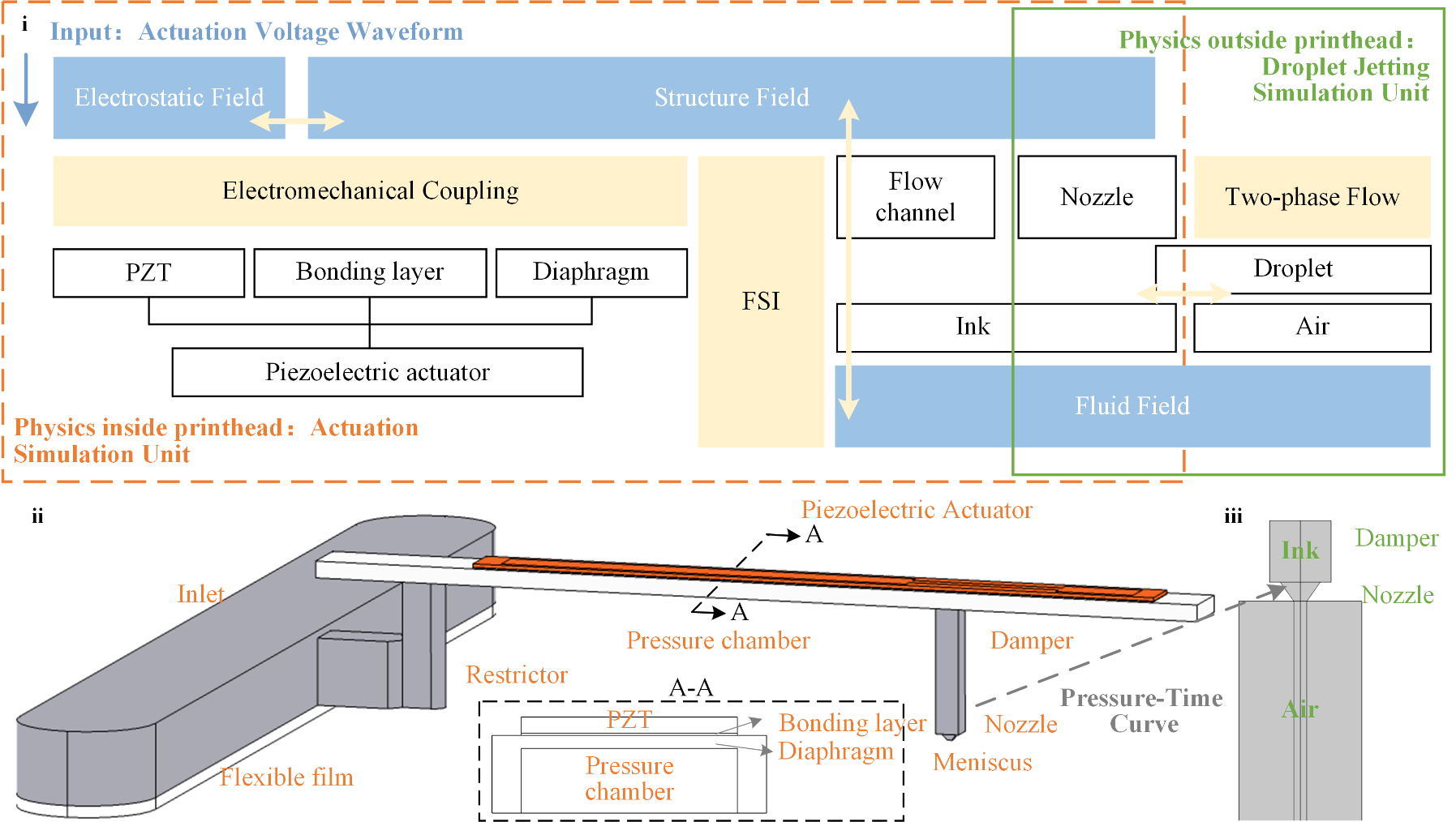


**Fig. S15** Schematic of the numerical simulation model

## *Fig. S16.*


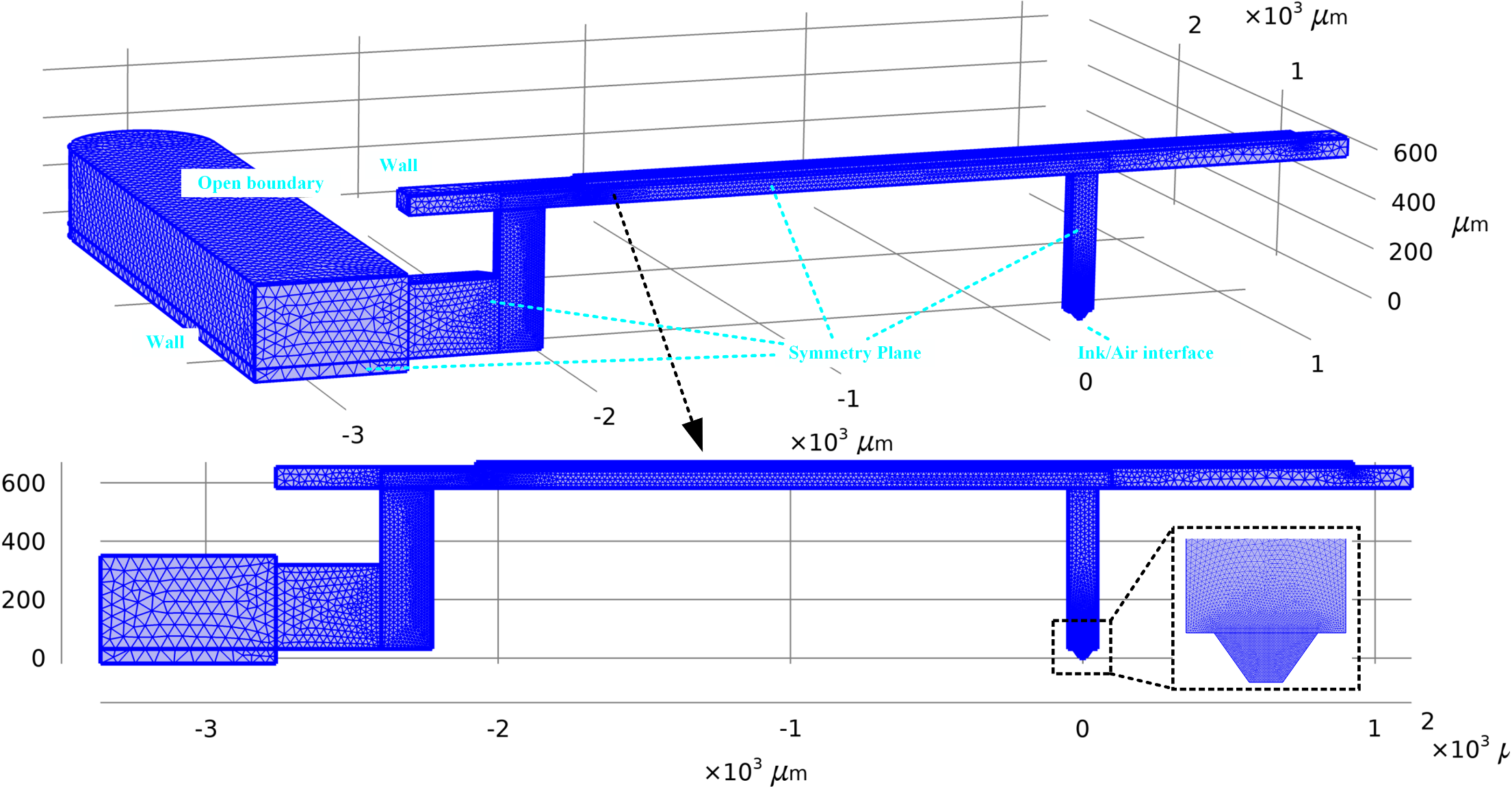


**Fig. S16.** The boundary conditions and the mesh division of actuation simulation unit.

## *Fig. S17.*


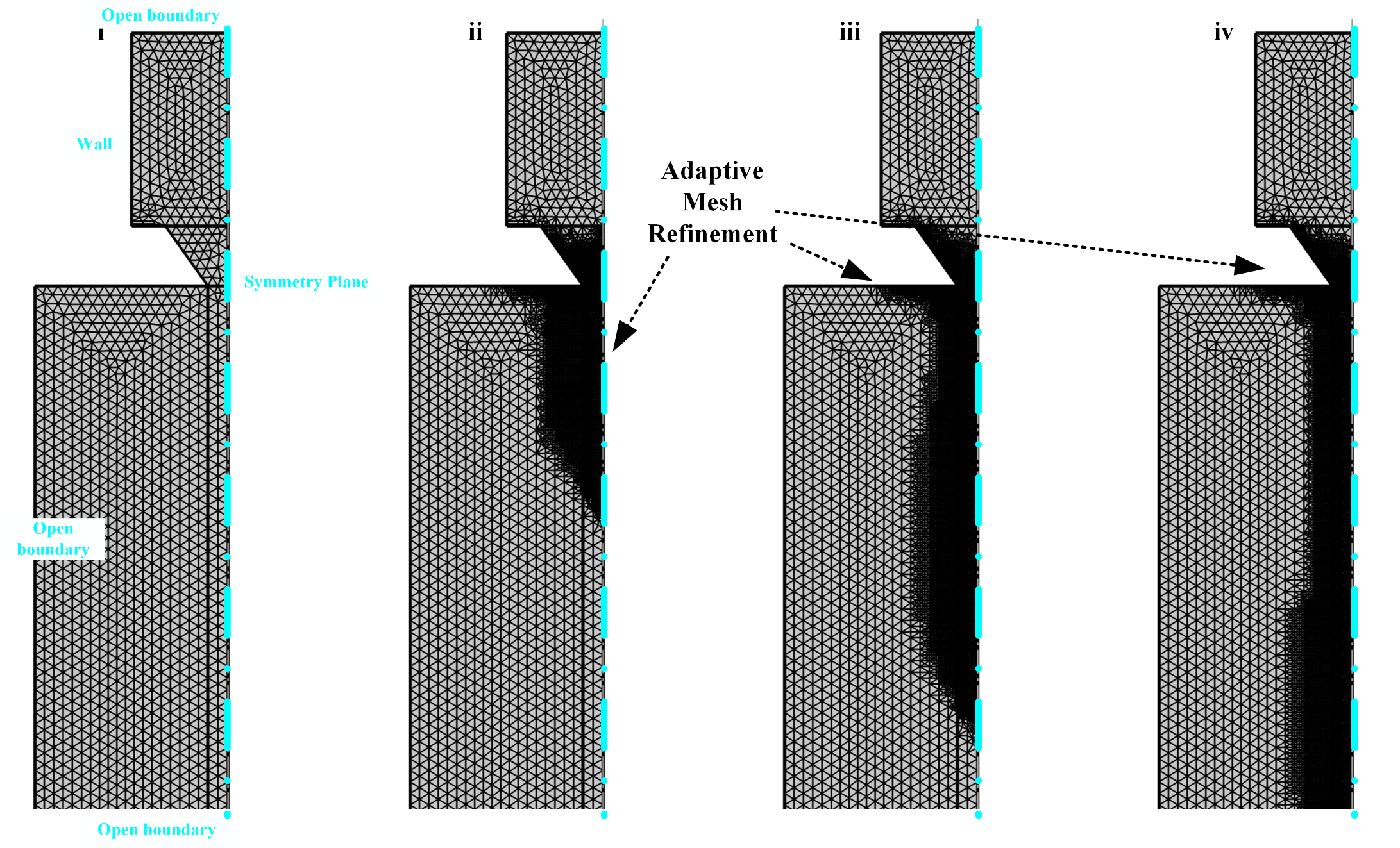


**Fig. S17.** The boundary conditions and the mesh division of droplet jetting simulation unit.

## *Fig. S18.*

**
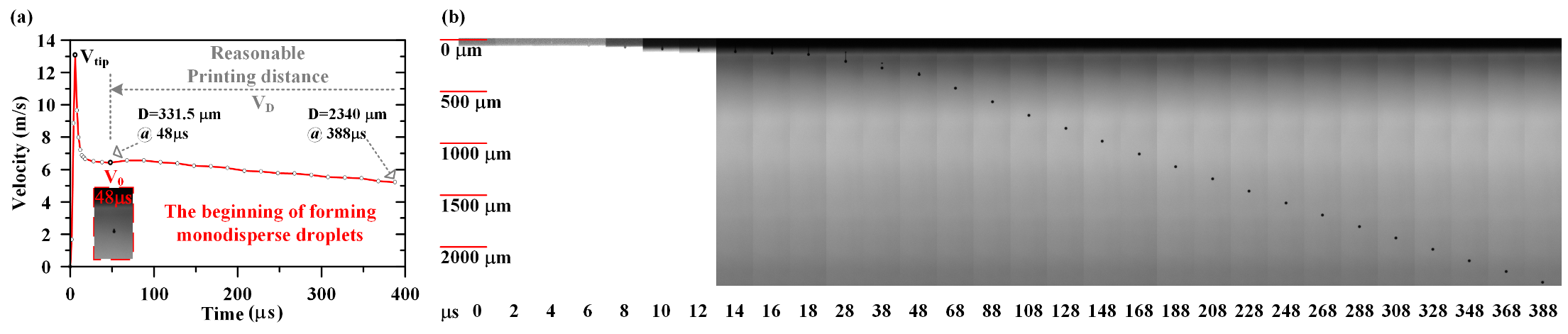
**

**Fig. S18.** (a) The typical droplet velocity variation process. (b) Experimental images of droplet jeting process.

## *Fig. S19.*


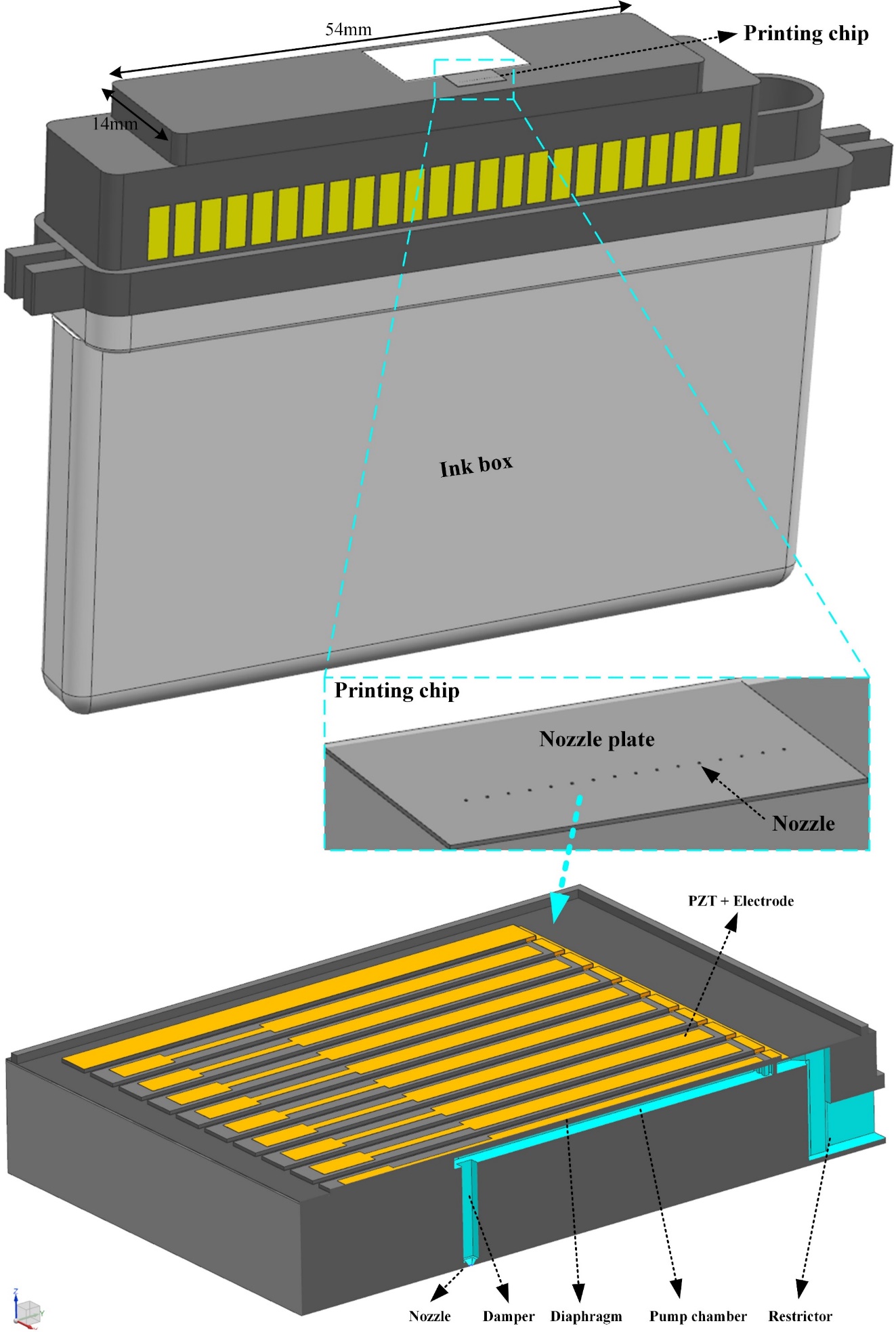


**Fig. S19.** Schematic diagram of the DMC-11610 printhead.

## *Table S1.*

**Table S1.** The peak values and peak arrival time under different T_l_.

| T_l_ | A_0_ | | B_0_ | | A_1_ | |
| --- | --- | --- | --- | --- | --- | --- |
|  | Value  (kPa) | Time  (μs) | Value  (kPa) | Time  (μs) | Value  (kPa) | Time  (μs) |
| 1.1  0.8  0.5  0.2 | 49.54  53.37  55.13  55.23 | 1.1  0.8  0.5  0.4 | -34.47  -35.13  -35.41  -35.27 | 3.9  3.8  3.6  3.5 | 19.80  19.82  20.16  19.91 | 7.4  7.3  7.1  7 |

## *Table S2.*

**Table S2.** The peak values and peak arrival time under different waveforms.

| Waveform | | Waveform b-i | Waveform b-ii | Waveform b-iii | Waveform b-iv |
| --- | --- | --- | --- | --- | --- |
| A_0_ | Value (kPa) | 57.42 | 57.42 | 57.42 | 57.42 |
|  | Time (μs) | 0.3 | 0.3 | 0.3 | 0.3 |
| B_0_ | Value (kPa) | -35.31 | -90.25 | -90.25 | -90.25 |
|  | Time (μs) | 3.6 | 3.9 | 3.9 | 3.9 |
| A_1_ | Value (kPa) | 19.85 | 55.33 | 112.82 | 112.82 |
|  | Time (μs) | 7 | 6.9 | 7.2 | 7.2 |
| B_1_ | Value (kPa) | - | -30.01 | -62.11 | -111.49 |
|  | Time (μs) | - | 10.4 | 10.5 | 10.7 |
| A_2_ | Value (kPa) | - | - | 36.94 | 141.00 |
|  | Time (μs) | - | - | 13.8 | 14.1 |
| B_2_ | Value (kPa) | - | - | - | -137.89 |
|  | Time (μs) | - | - | - | 17.6 |
| A_3_ | Value (kPa) | - | - | - | 165.23 |
|  | Time (μs) | - | - | - | 21 |
| B_3_ | Value (kPa) | - | - | - | -162.39 |
|  | Time (μs) | - | - | - | 24.4 |
| A_4_ | Value (kPa) | - | - | - | 181.30 |
|  | Time (μs) | - | - | - | 27.9 |
| B_4_ | Value (kPa) | - | - | - | -178.49 |
|  | Time (μs) | - | - | - | 31.3 |
| A_5_ | Value (kPa) | - | - | - | 187.01 |
|  | Time (μs) | - | - | - | 34.8 |
| B_5_ | Value (kPa) | - | - | - | -184.03 |
|  | Time (μs) | - | - | - | 38.2 |
| A_6_ | Value (kPa) | - | - | - | 186.68 |
|  | Time (μs) | - | - | - | 41.7 |
| B_6_ | Value (kPa) | - | - | - | -183.89 |
|  | Time (μs) | - | - | - | 45.1 |

## *Table S3.*

**Table S3.** Statistics and comparisons of the improvements in printing speed and printing distance.

| Size | Classical  PIJ method | | UHDV  PIJ method | | Improvement | |
| --- | --- | --- | --- | --- | --- | --- |
|  | Printing  speed (m/s) | Printing distance (mm) | Printing  speed (m/s) | Printing distance (mm) | Magnification of printing speed | Magnification of printing distance |
| DMC-11601 printhead  (9 μm nozzle) | 0.05 | 1.5 | 2.5 | 6.5 | 50 times | 4.33 times |
|  | 0.1 | 1.25 |  |  | 25 times | 5.20 times |
|  | 0.2 | 0.7 |  |  | **12.5 times** | **9.29 times** |
| DMC-11610 printhead  (21 μm nozzle) | 0.05 | 4 | 2.5 | 17 | 50 times | 4.25 times |
|  | 0.1 | 3 |  |  | 25 times | 5.67 times |
|  | 0.2 | 2.5 |  |  | 12.5 times | 6.8 times |
|  | 0.25 | 2 |  |  | 10 times | 8.5 times |

## *Table S4.*

**Table S4.** Overall comparision between the potential of the proposed UHDV-PIJ and the classical PIJ methods.

| Number | Parameters | This work | Classical PIJ methods |
| --- | --- | --- | --- |
| i | Typical maximum droplet velocity | a few m/s to >20 m/s @1 mm (27.53 m/s max, for the ink viscosity of 5.1 cp to 25.2 cp). | 5-8 m/s @ 1 mm (>10 m/s or even a little higher for low ink viscosity of a few cp). |
| ii | Typical maximum printing distance | theoretically at the cm level;  experimental test data: 6.5 mm for 9 μm nozzle and 17 mm for 21 μm nozzle (under a maximum printing speed of 2.5 m/s). | theoretically at a few mm (0.5-2 mm);  experimental test data: 1.5 mm for 9 μm nozzle and 4 mm for 21 μm nozzle (under minimum printing speed of 0.05 m/s). |
| iii | Typical maximum printing speed | theoretically at the m/s level;  experimental test data: 2.5 m/s for 9 μm nozzle and 21 μm nozzle under the maximum printing distances of 6.5 mm and 17 mm, respectively. | theoretically at tens to hundreds of mm/s (<0.5 m/s);  experimental test data: 0.2 m/s for 9 μm nozzle and 0.25 m/s for 21 μm nozzle under the minimum printing distances of 0.7 mm and 2 mm, respectively. |
| iv | Droplet state at high velocity | satellite free. | satellite droplets (almost inevitable). |
| v | The compatibility of ink materials | experimental test data: Oh number: 0.03-1.18; Viscosity: 1-40.3 cp; Surface tension: 64.1-72.2 mN/m. | theoretical Oh number: 0.1-1;  manufacturer's recommended data: Oh number: 0.36-0.67; Viscosity: 10-12 cp; Surface tension: 32-42 mN/m. |

## *Table S5.*

**Table S5.** Physical properties of the glycerine-water inks.

| Serial Number | Glycerine percent  weight (%) | Viscosity at 25 ℃  μ (mPa·s) | Surface tension at 25 ℃  σ (mN/m) | Density at 25 ℃  ρ (kg/m^3^) |
| --- | --- | --- | --- | --- |
| I  II  III  IV  V  VI  VII | 0  50  60  65  70  75  80 | 1.0  5.1  9.2  12.5  17.9  25.2  40.3 | 72.2  67.9  66.3  66.0  65.6  64.9  64.1 | 998.0  1119.3  1149.1  1162.3  1177.9  1190.3  1206.6 |

## *Table S6.*

**Table S6.** Geometric parameters of main components of the piezoelectric printhead

| Components | Parameters value |
| --- | --- |
| PZT | 16 μm×200 μm×3000 μm |
| Membrane | Thickness 10 μm |
| Top electrode | 2 μm×140 μm×1780 μm |
| Bottom electrode | 2 μm×140 μm×3000 μm |
| Pump chamber | 60 μm×200 μm×2320 μm |
| Inlet | 360 μm×200 μm×288 μm |
| Restrictor | 125 μm×100 μm×551 μm |
| Damper | 100 μm×100 μm×551 μm |
| Nozzle | Top side length 65 μm  Bottom side length 21 μm  Height 31 μm |
| Flexible film | Thickness 50 μm |

## *Table S7.*

**Table S7.** The physical properties of the materials used in the simulation model

| Components | Materials | Parameters value | |
| --- | --- | --- | --- |
| PZT | PZT | Piezoelectric constant  d_31_ (10^-10^ C/N) | -2.74 |
|  |  | Piezoelectric constant  d_33_ (10^-10^ C/N) | 5.93 |
|  |  | Piezoelectric constant  d_15_ (10^-10^ C/N) | 7.41 |
|  |  | Density (kg/m^3^) | 7500 |
|  |  | Poisson’s ratio | 0.35 |
|  |  | Young’s modulus (10^9^ Pa) | 67 |
| Electrode | Au | Poisson’s ratio | 0.44 |
|  |  | Young’s modulus (10^9^ Pa) | 70 |
| Wall and Diaphragm | Si | Poisson’s ratio | 0.28 |
|  |  | Young’s modulus (10^9^ Pa) | 170 |
| Flexible film | Teflon | Young’s modulus (10^9^ Pa) | 0.4 |

## *Supplementary References:*

[R1] Lohse. D., Fundamental fluid dynamics challenges in inkjet printing, Annu. Rev. Fluid Mech 54 (2022) 349-382. https://doi.org/10.1146/annurev-fluid-022321-114001

[R2] Derby. B., Inkjet printing of functional and structural materials: fluid property requirements, feature stability, and resolution, Annu. Rev. Mater. Res 40 (2010) 395-414. https://doi.org/10.1146/annurev-matsci-070909-104502

[R3] Gareth H. M., Michael R., 2011.Wolfgang von Ohnesorge. Phys. Fluids., 23 (12), 127101.

https://doi.org/10.1063/1.3663616

[R4] Derby. B., 2015. Additive manufacture of ceramics components by inkjet printing. Engineering, 1(1), 113-123. https://doi.org/10.15302/J-ENG-2015014

[R5] FUJIFILM Dimatix Materials Printer DMP-2800 Series, User Manual, www.dimatix.com.

[R6] T. Tao, Moving mesh methods for computational fluid dynamics. in recent advances in adaptive computation, Contemp. Math. 383 (2005) 141–173, https://doi.org/10.1090/conm/383/07162

[R7] Fulton. A., Drop volume modulation via modulated contact angle in inkject systems, Purdue University, 2014.

[R8] Hoath. S. D., Hsiao. W. K., Jung. S., Martin. G. D., Hutchings. I. M., Morrison. N. F., Harlen. O. G., 2013. Drop speeds from drop-on-demand ink-jet print heads. Journal of Imaging Science and Technology, 57(1) 010503. https://doi.org/10.2352/J.ImagingSci.Technol.2013.57.1.010503.
